# *Lactobacillus gasseri* extracellular proteins suppress acne inflammation and sebum secretion via the PI3K/AKT/mTOR pathway

**DOI:** 10.1186/s40643-026-01074-9

**Published:** 2026-06-01

**Authors:** Zixin Song, Qinxuan Yuan, Yifan Fang, Bianbian Zhai, Jiman Geng, Meng Li, Changtao Wang, Dongdong Wang

**Affiliations:** School of Light Industry Science and Engineering, Beijing Technology and Business University, Fucheng Road, Beijing, 100048 China

**Keywords:** Extracellular proteins, Acne, PI3K/AKT/mTOR pathway, Anti-inflammatory

## Abstract

**Abstract:**

This study evaluated the anti-acne potential of extracellular proteins derived from *Lactobacillus gasseri* (LG-EPs) by integrating physicochemical characterization, antibacterial assessment, cellular assays, and an in vivo sebaceous gland model. LG-EPs were obtained by ammonium sulfate precipitation and characterized using gel permeation chromatography/light scattering (GPC/LS) and liquid chromatography–tandem mass spectrometry (LC–MS/MS). The molecular weight of LG-EPs was mainly distributed between 1.0 × 10⁴ and 2.0 × 10⁴ g/mol, and peptide analysis identified four peptides with antimicrobial potential and twelve peptides with antioxidant potential. LG-EPs exhibited direct antibacterial activity against acne-associated bacteria, with MIC and MBC values of 600 µg/mL against *Cutibacterium acnes* and MIC and MBC values of 700 µg/mL and 1 mg/mL, respectively, against *Staphylococcus aureus*. Growth-curve and biofilm adhesion assays further showed that LG-EPs inhibited bacterial proliferation and adhesion. In LPS-stimulated HaCaT cells, LG-EPs reduced the secretion of pro-inflammatory cytokines, including IL-6, IL-8, IL-1β, and TNF-α, while increasing the expression of barrier-related factors such as AQP3, FLG, and LOR. In a golden hamster model, topical LG-EP treatment decreased sebum production and downregulated the transcription of lipogenesis-related genes, including SREBP-1, FAS, and ACC1. These effects were associated with reduced transcript levels of PI3K, AKT, and mTOR, while the anti-sebum effect was accompanied by increased AMPK transcription. Overall, LG-EPs showed multi-target anti-acne potential through antibacterial, anti-inflammatory, barrier-protective, and sebum-suppressive effects.

**Graphical abstract:**

## Introduction

*Lactobacillus gasseri* is a gram-positive, facultatively anaerobic lactic acid bacterium and one of the most common types of strains that lives in the human small intestine (Ying Zhang et al. 2023a). It can also be extracted from various parts of the body, such as the oral cavity, gastrointestinal tract, vagina, and areola, and feces (Zorič Peternel et al. 2010). *L. gasseri* has been verified to be appropriate for use in food as an additive since the U.S. Food and Drug Administration classified it as a generally recognized as safe (GRAS) microorganism. This bacteria is a significant probiotic. It can withstand acidic environments, has a very strong affinity for epithelial cells and is capable of producing antimicrobial bacteriocins on pathogens(Xu et al. 2020). In addition, various uses of *L. gasseri* have been demonstrated in the food and medical sectors, including in the alleviation of allergic disorders, oxidative stress regulation, and control of inflammatory conditions(Huang et al. 2025).

The promise of *L. gasseri* and other probiotics has prompted a search to determine the role of these probiotics in common inflammatory diseases, including acne. The presence of sebaceous glands with hair follicles can lead to acne (Platsidaki and Dessinioti 2018). There are four main factors involved in its pathogenesis (Goldberg et al. 2023): overcolonization of *Cutibacterium acnes*, excessive production of sebum, excessive hair follicle hyperkeratosis, and inflammation. Acne may further develop into erythema, hyperpigmentation and hypertrophic scars in the event of unattended conditions, severely impacting the psychological status of patients. The existing treatment methods for acne (e.g., antibiotics) may result in negative outcomes, such as redness, swelling and burns, and promote antimicrobial resistance (Sheffer-Levi et al. 2020). These findings indicate that new treatment methods are urgently needed, and recent investigations have indicated that different probiotics are useful for the treatment of acne because they alleviate inflammation and reduce the number of acne-inducing bacteria (Cui et al. 2022).

Research is beginning to focus on the functional metabolites of live probiotics, which are commonly referred to as post-biotics. Compared with parent cells, the extracellular proteins (EPs) or secretome of bacteria that are released as a postbiotic are safer and more stable. Postbiotics are more stable to store and have greater potential for use since they are no longer viable and present no risk of infection or horizontal genetic transfer (Narli and Ozcan 2022). Compared with probiotics, prebiotics are safer and more stable and exhibit greater storage stability and greater potential for application (Ooi and Liong 2010).

The sebaceous gland is an important factor in the pathogenesis of acne. Overexpression of sebaceous glands and hypersecretion of sebum significantly contribute to the development of acne (Shamloul and Khachemoune 2021). In this way, the downregulation of sebaceous gland activity and the control of sebum secretion are among the major methods for managing acne (Makrantonaki et al. 2011). In golden hamsters, sebaceous plaque areas that are bilaterally placed on the dorsal surface provide a natural in vivo model, which can be assessed using sebum-regulating interventions. Androgens regulate the area and pigmentation of coarse hairs, their thickness, and the cells of sebaceous glands as well as melanocytes(Weissmann et al. 1984). SREBP-1 and acetyl-CoA carboxylase (ACC) are involved in steroid production and fatty acid production, respectively (Heo et al. 2020). Both are essential for the metabolism and fatty acid development of microorganisms, and SREBP-1 is an important transcription element that increases the expression of ACC (Melnik 2017). As a result, assessment the effects of the tested samples on sebum secretion is possible by determining the difference in the expression of the SREBP-1 and ACC genes.

Several upstream regulators affect the mTOR signaling pathway, including the AMPK, PI3K/AKT, MAPK, and p53 pathways (Vander Broek et al. 2015). The PI3K/AKT/mTOR axis is among the best-conserved signaling cascades that play a central role in the regulation of cell proliferation, oxidative stress, and inflammation (Luo et al. 2022). Previous research has shown that mTOR is overexpressed in the cytoplasm and nucleus of inflammatory sebaceous glands in acne lesions compared with nonlesional skin (Inoue et al. 2014).

**Fig. 1:**
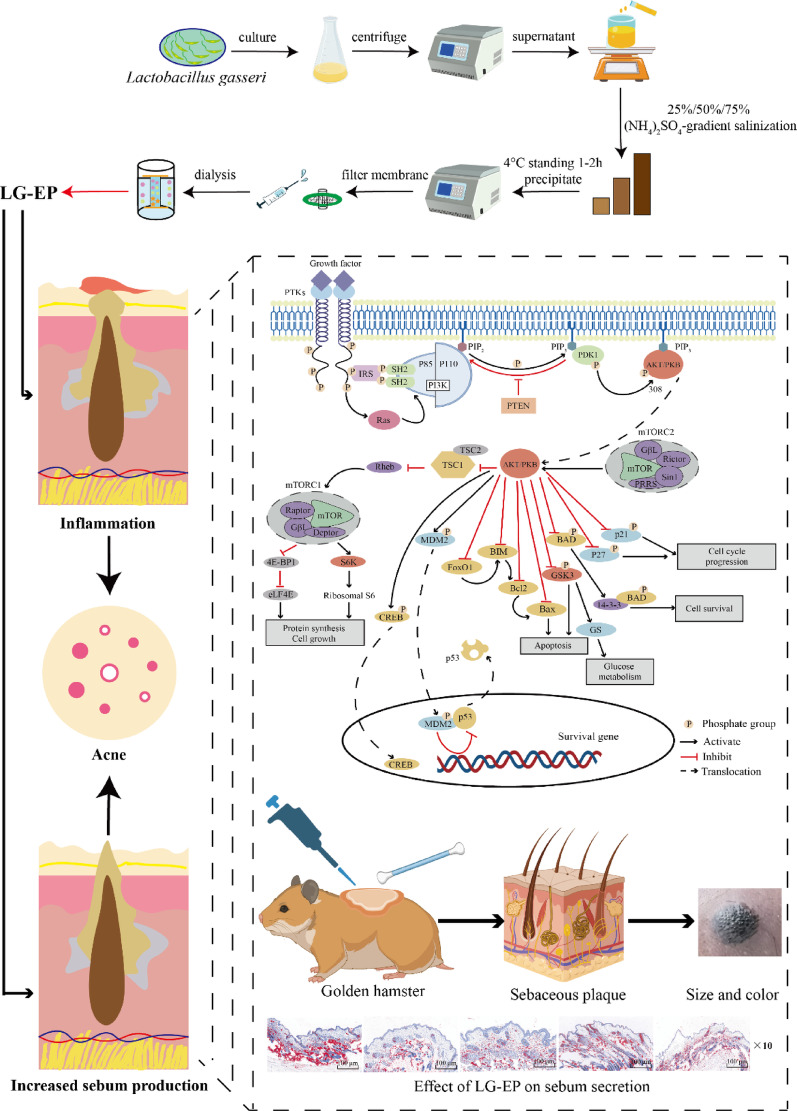
LG-EPs effectively treat acne by reducing inflammation and inhibiting sebum production

As shown in Fig. 1, in the present study, extracellular proteins of *Lactobacillus gasseri* (LG-EPs) were isolated using ammonium sulfate gradient salt-out. Their molecular weight and peptide sequences were first characterized through gel permeation chromatography/light scattering (GPC/LS) and liquid chromatography–tandem mass spectrometry (LC–MS/MS). The anti-inflammatory properties of LG-EPs were subsequently assessed by establishing an LPS-induced inflammatory model in HaCaT cells. Finally, the effects of LG-EPs on sebum secretion were examined in animal experiments. Collectively, the findings provide theoretical and experimental support for the potential application of LG-EPs in anti-acne treatment.

## Materials and methods

### Materials and chemicals

*Lactobacillus gasseri* was purchased from the China Industrial Microbial Culture Collection Center. 1,1-diphenyl-2-picryl-hydrazyl radical (Nanjing Jiancheng Bioengineering Institute); ferrous sulfate, hydrogen peroxide, salicylic acid, anhydrous sodium carbonate, isopropanol, and neutral gum (Sinopharm Group); anhydrous ethanol (Beijing Chemical Industry Group Co.); lipopolysaccharide (Biorigin (Beijing) Inc.); HaCaT cell (Cell Resource Center, Beijing Union Medical College); Dulbecco’s modified Eagle’s medium (Thermo Fisher Scientific Inc.); phosphate buffer saline; cell counting kit-8; ROS assay kit; ELISA kits (Biorigin (Beijing) Inc.); PMSF; western blot IP cell lysate (Shanghai Biyuntian Biotechnology Co.); 6-well and 96-well plates (Corning); FFA content test kits (Beijing Solarbio Technology Co.); TG test kits (Biorigin (Beijing) Inc.); and Oil Red O dye solution and HE dye solution (Wuhan Servicebio Biotechnology Co.) were used.

### Preparation of *Lactobacillus gasseri* extracellular proteins (LG-EPs)

#### Preparation of *Lactobacillus gasseri* fermentation broth

One hundred grams of MRS medium and 2 g of glucose were weighed and then dissolved in 300 mL of deionized water. The mixture was sterilized at 121 °C for 30 min and allowed to cool. Once cooled, the medium were inoculated with 0.1% *Lactobacillus gasseri*, and a constant temperature of 43 °C was maintained for static fermentation for 48 h. After fermentation was complete, the mixture was centrifuged at 4800 rpm for 30 min to obtain the supernatant, which was the *Lactobacillus gasseri* fermentation broth.

#### Preparation of extracellular proteins from *Lactobacillus gasseri*

The *Lactobacillus gasseri* fermentation broth was filtered through a 0.22-µm filter. The sterile fermentation supernatant was collected, and the supernatant was subjected to protein salting out on a magnetic stirrer (25 °C, 200 rpm) using the ammonium sulfate gradient salting out method (25%-50%-75%). After 1 h, the supernatant was allowed to stand for 2 h in a refrigerator at 4 °C and centrifuged for 15 min at 4 °C and 1200 rpm to collect the supernatant and protein precipitate. The supernatant was then subjected to continuous salting out, and the above steps were repeated until the three gradients were completed. The protein precipitate was dissolved in Tris-HCl at pH = 7.5, passed through a 0.22-µm filter membrane, placed in a 500-Da dialysis bag, and dialyzed. The liquid was changed 2–3 times a day for 3–4 d, and the final *Lactobacillus gasseri* extracellular proteins, LG-EPs, were obtained.

### Determination of the molecular weight of LG-EPs using the GPC/LS method

The molecular weight of LG-EPs was determined via the GPC/LS method.

### Identification of peptides in the LG-EPs using LC‒MS/MS

Peptide sequence analysis was carried out by Beijing BiotechPack Co. Briefly, 1 mL of sample was transferred to a 10-kDa ultrafiltration tube and centrifuged at 12,000 × g for 10 min at 4 °C to collect the < 10 kDa fraction. The filtrate was reduced with dithiothreitol (DTT, final concentration 10 mM) at 56 °C for 1 h, cooled to room temperature, and alkylated with iodoacetamide (IAM, final concentration 20 mM) in the dark for 40 min. Excess IAM was quenched with DTT to a final concentration of 10 mM. Peptides were desalted using C18 StageTips and vacuum-dried at 45 °C before LC‒MS/MS analysis.

Liquid chromatography conditions: analytical column, 75 μm i.d. × 25 cm NanoViper C18 (1.9 μm, 100 Å); mobile phase A, 0.1% trifluoroacetic acid (TFA) in water; mobile phase B, 0.1% TFA in 80% acetonitrile; flow rate, 600 nL/min; total run time, 35 min.

Elution gradient: 0–2 min, 4% B; 2–8 min, 4% B; 8–20 min, 4–32% B; 20–23 min, 32–55% B; 23–25 min, 55–95% B; 25–30 min, 95% B; 30–31 min, 95–4% B; 31–35 min, 4% B.

The mass spectrometry parameters were as follows: MS1 resolution, 60,000 (at m/z 200); AGC target, standard; maximum injection time (Max IT), 50 ms; MS2 resolution, 15,000 (at m/z 200); AGC target, standard; Max IT, cycle time 2 s.

### Safety determination

#### Rabbit erythrocyte hemolysis (RBC) assay

Fresh rabbit blood was centrifuged at 1500×g for 10 min, and the precipitate and PBS were removed and diluted at a ratio of 1:49. Blood washing was completed when the absorbance value at 541 nm was 0.5 ± 5% after 0.5 mL of cell suspension and 4.5 mL of deionized water were mixed well. In this experiment, deionized water was used as a positive control (PC), and PBS was used as a negative control (NC). The cell suspension was mixed with different concentrations of samples, deionized water, and PBS at a ratio of 1:3, and the total volume was adjusted to 1 mL. The cells were incubated for 60 min in a shaker at room temperature and then centrifuged for 1 min at 10,000×g to terminate the incubation, after which the absorbance value at 560 nm was determined, and the hemolysis rate of the samples was calculated according to the formula.

$$ \begin{gathered} {\text{Hemolysis rate}}\% {\text{ }} = \left( {{\mathrm{OD56}}0{\text{ sample group }} - {\text{ OD56}}0{\text{ negative control group}}} \right)/ \hfill \\ \left( {{\mathrm{OD56}}0{\text{ positive control group }} - {\text{ OD56}}0{\text{ negative control group}}} \right) \times {\mathrm{1}}00\% \hfill \\ \end{gathered} $$

#### Chick embryo chorionic allantoic membrane eye irritation test (HET-CAM)

The fertilized eggs were incubated in an incubator at a temperature of 38 °C and a humidity of 60%-70% for 9–10 days. The position of the gas chamber was checked with a torch, and the eggshell was peeled off with tweezers to expose the white eggshell membrane in the gas chamber. Five hundred microliters of 0.9% NaCl solution was added to infiltrate the membrane of the eggshell, and the membrane of the eggshell was carefully torn off with tweezers to expose the blood vessels required for the experiment. In this experiment, 0.9% NaCl solution was used as a negative control, and 0.1 mol/L NaOH was used as a positive control. Three hundred microliters of the sample, negative control, or positive control were added dropwise to the peeled eggs, the vascular damage was observed for 3 min, and the changes in vascular damage before and after sample addition were recorded. The results of this experiment were evaluated using the endpoint evaluation (ES) method (Table 1), where six sets of parallel experiments were performed for each sample, and the samples were categorized according to the ES values according to the table (Table 2) for eye irritation.

**Table 1:** Observation scoring

Type of Damage	Score	Grade	Appearance
Bleeding	0	–	No bleeding
	1	Mild	Bleeding from small blood vessels and small amounts of hemorrhage only
	2	Moderate	Bleeding from small and large blood vessels with a significant amount of blood coming out
	3	Serious	Bleeding from almost all the veins, a lot of blood coming out
Clotting	0	–	Noncoagulable
	1	Mild	Mild intra/extravascular coagulation, mild clouding of CAM membranes
	2	Moderate	Moderate intra/extravascular coagulation, moderate clouding of CAM membranes
	3	Serious	Heavy intra/extravascular coagulation with heavy clouding of CAM membranes
Vascular lysis	0	–	Nonvascularized lysis
	1	Mild	Small vessel lysis only
	2	Moderate	Small and large vessel lysis
	3	Serious	Lysis of the great vessels and all the vascular trees

**Table 2:** Evaluation of the results of the endpoint scoring method

Endpoint rating	Irritation classification
ES ≤ 12	No/mild irritation
12< ES<16	Moderate irritation
ES ≥ 16	Strongly irritating/corrosive

### Antibacterial activity of LG-EPs against acne-associated bacteria

#### Bacterial strains and culture conditions

*Staphylococcus aureus* and *Cutibacterium acnes* were obtained from the China General Microbiological Culture Collection Center. *S. aureus* was activated by inoculating 1% of the bacterial stock into LB liquid medium and culturing it at 37 °C with shaking at 120 rpm for 24 h. *C. acnes* was activated by inoculating 1% of the bacterial stock into RCM liquid medium and culturing it anaerobically at 37 °C for 48 h. The bacterial suspensions were then diluted with LB or RCM medium to 1 × 10^8 CFU/mL (OD600 = 0.08–0.13), followed by a 100-fold dilution to obtain a final working concentration of 1 × 10^6 CFU/mL for subsequent antibacterial assays.

#### Determination of MIC and MBC

Different concentrations of LG-EPs were prepared in LB or RCM liquid medium. For *S. aureus*, each well of a 96-well plate was filled with 100 µL of bacterial suspension (1 × 10^6 CFU/mL) and 100 µL of LG-EP solution at different concentrations, followed by incubation at 37 °C for 24 h. The minimum inhibitory concentration (MIC) was defined as the lowest concentration showing no visible bacterial growth. The cultures were then further incubated for another 24 h, and the minimum bactericidal concentration (MBC) was defined as the lowest concentration at which no bacterial colony was observed.

For *C. acnes*, the same procedure was performed using RCM medium under anaerobic conditions at 37 °C for 48 h. The MIC was defined as the lowest concentration at which no visible bacterial growth was observed, and the MBC was defined as the lowest concentration at which no bacterial colony appeared after further incubation for 24 h.

#### Growth curve assay

The effects of LG-EPs on the growth curves of *S. aureus* and *C. acnes* were evaluated according to a previously reported method with minor modifications. Briefly, 600 µL of *S. aureus* or *C. acnes* culture in the logarithmic growth phase was inoculated into 30 mL of LB or RCM liquid medium (2% inoculum). LG-EPs were added to achieve the MIC concentration, while bacterial medium without LG-EPs served as the blank control (BC). The initial absorbance at 600 nm was recorded as the 0 h time point.

The *S. aureus* cultures were incubated at 37 °C with shaking at 180 rpm, and the OD600 values were recorded every 2 h. The *C. acnes* cultures were incubated anaerobically at 37 °C for 48 h, and the OD600 values were recorded every 4 h. Growth curves were plotted with OD600 as the ordinate and time as the abscissa.

#### Biofilm adhesion assay

The effects of LG-EPs on biofilm adhesion of *S. aureus* and *C. acnes* were determined using the crystal violet staining method according to a previously reported method with slight modifications (Yongtao Zhang et al. 2023b). Briefly, each well of a 48-well plate was filled with 200 µL of bacterial suspension (1 × 10^6 CFU/mL) and 200 µL of LG-EP solution at concentrations of 1/2 MIC, MIC, and 2 MIC. The positive control group received 200 µL of erythromycin (30 µg/mL), whereas the negative control group received 200 µL of sterile water.

The *S. aureus*-inoculated plates were incubated at 37 °C for 24 h, whereas the *C. acnes*-inoculated plates were incubated anaerobically at 37 °C for 48 h. After incubation, the supernatants were discarded, and the wells were gently washed twice with 500 µL of sterile water. The adherent biofilms were fixed with 500 µL of 25% glutaraldehyde at room temperature for 15 min. After fixation, the wells were washed twice with sterile water and stained with 200 µL of 0.1% crystal violet for 5 min. The crystal violet solution was removed, and the wells were washed twice again with sterile water. The biofilm morphology was observed under an inverted microscope. Finally, the adherent bacteria were dissolved in anhydrous ethanol, and the absorbance at 590 nm was measured. The inhibition rate of adhesion was calculated as follows:

$$ \begin{gathered} {\text{Inhibition rate }}\left( \% \right){\text{ }} = {\text{ }}\left( {{\mathrm{OD59}}0{\text{ of negative control group }} - {\text{ OD59}}0{\text{ of sample group}}} \right) \hfill \\ {\text{ }}/{\text{ OD59}}0{\text{ of negative control group }} \times {\text{ 1}}00\% \hfill \\ \end{gathered} $$

### Determination of antioxidant and anti-inflammatory activities in vitro

#### DPPH free radical scavenging assay

Four milligrams of DPPH was accurately weighed and dissolved in 50 mL of anhydrous ethanol. This solution was subsequently mixed with deionized water at a 1:1 ratio and was used when the absorbance value at 517 nm exceeded 0.7. Three groups were established: the sample group, the control group, and the blank group. Six concentrations of the sample were prepared: 31.25, 62.5, 125, 250, 500 and 1000 µg/mL. The experiments were conducted according to the spiking method outlined in Table 3. After the reaction was allowed to proceed for 30 min in the dark, the absorbance values were measured at 517 nm, and the scavenging rates of the samples for DPPH radicals were calculated using the appropriate formula.

$$ {\text{DPPH radical scavenging rate}}\% {\text{ }} = {\text{ }}\left[ {\left( {{\mathrm{A}}_{{\mathrm{2}}} - {\mathrm{A}}_{{\mathrm{1}}} + {\mathrm{A}}_{{\mathrm{3}}} } \right)/{\mathrm{A}}_{{\mathrm{2}}} } \right] \times {\mathrm{1}}00\% $$

**Table 3:** DPPH radical scavenging assay spiking sequence

Reagent	Volume (mL)		
	A_1_	A_2_	A_3_
DPPH solution	0.5	0.5	0.0
sample	0.5	0.0	0.5
deionized water	0.0	0.5	0.5

#### Hydroxyl radical scavenging assay

The scavenging effect of the samples on hydroxyl radicals was evaluated using the salicylic acid method. Solutions of 8.0 mmol/L FeSO_4_, 3.0 mmol/L salicylic acid, and 0.02 mol/L H_2_O_2_ were prepared. The experimental design included sample, blank, and sample background groups, with the samples prepared at six concentrations: 31.25, 62.5, 125, 250, 500, and 1000 µg/mL. The experiments were conducted according to the spiking method outlined in Table 4, with the reaction occurring in a water bath at 37 °C for 1 h. Following the reaction, the samples were centrifuged at 4000 rpm for 10 min, after which the supernatants were collected. The absorbance at 510 nm was measured to calculate the scavenging rate of hydroxyl radicals by the samples using the formula provided below.

$$ {\text{Hydroxyl radical scavenging rate}}\% {\text{ }} = {\text{ }}\left[ {\left( {{\mathrm{A}}_{0} - {\mathrm{A}}_{{\mathrm{1}}} + {\mathrm{A}}_{{\mathrm{2}}} } \right)/{\mathrm{A}}_{0} } \right] \times {\mathrm{1}}00\% $$

**Table 4:** Hydroxyl radical scavenging assay spiking sequence

Reagent	Volume (mL)		
	A_0_	A_1_	A_2_
8 mmol/L FeSO_4_	0.3	0.3	0.3
3 mmol/L salicylic acid	1.0	1.0	0.0
sample	0.0	1.0	1.0
deionized water	1.45	0.45	1.45
0.02 mol/L H_2_O_2_	0.25	0.25	0.25

#### Hyaluronidase inhibition assay

Hyaluronidase activity was assessed using the modified Reissig fluorescence Morgan‒Elson method, in accordance with the specific experimental procedures detailed in the literature (Takahashi et al. 2003).

2.8 Modeling of LPS damage, screening of the optimal action concentration and determination of the ROS concentration.

HaCaT cells were recovered from frozen storage by thawing the cryovials in a 37 °C water bath, followed by transfer into 9 mL of complete DMEM containing 10% fetal bovine serum and 1% penicillin–streptomycin. After centrifugation at 1500 rpm for 5 min, the supernatant was discarded, and the pellet was resuspended in 1 mL of complete DMEM. The resulting suspension was then seeded into a T25 culture flask prefilled with 5 mL of complete medium and maintained at 37 °C in a 5% CO₂ incubator.

To establish a lipopolysaccharide (LPS)-induced inflammatory model, interleukin-6 (IL-6) and interleukin-8 (IL-8) were selected as evaluation indices. HaCaT cells were plated into 6-well plates at a density of 5 × 10⁵ cells per well and incubated under the same conditions for 12 h. After the culture supernatant was removed, the cells were washed twice with PBS (1 mL per well). For the control group (C), 2 mL of serum-free DMEM was added, whereas the experimental groups were treated with 2 mL of LPS solutions at concentrations of 50, 100, 150, 200, and 250 µg/mL and incubated for 24 h. Supernatants were collected, and IL-6 and IL-8 levels were quantified using commercial ELISA kits according to the manufacturer’s protocols.

The influence of different sample concentrations on cell viability was examined using a CCK-8 assay. Briefly, 10 µL of CCK-8 reagent was added to each well, and the absorbance at 450 nm was measured following a 3 h incubation at 37 °C. Intracellular ROS levels were detected using the fluorescent probe DCFH-DA, following the procedures specified in the kit instructions.

### Enzyme-linked immunosorbent assay (ELISA)

HaCaT cells were seeded into 6-well plates at a density of 5 × 10⁵ cells per well and cultured for 12 h. The medium was then removed, and 2 mL of serum-free DMEM was added to the control group (C), while 2 mL of LPS solution (200 µg/mL) was added to the other wells. After 24 h of incubation, the supernatant was discarded, and serum-free DMEM was added to both the C and M groups. Test samples at various concentrations were subsequently added, followed by a 24-h incubation.

After treatment, the culture supernatant was collected and transferred to centrifuge tubes. The wells were rinsed with PBS, after which 200 µL of cell lysis buffer was added per well. Cells were detached using a cell scraper, and the lysates were transferred to centrifuge tubes. The samples were subsequently centrifuged at 10,000 rpm for 5 min, after which the supernatants were collected for analysis. The concentrations of IL-6, IL-8, IL-1β, TNF-α, AQP3, FLG, LOR, and caspase-14 were quantified using commercial ELISA kits in accordance with the manufacturer’s protocols.

### Animal experimentation

LVG golden hamsters (6–7 weeks old, male), SPF-grade, were acclimatized and fed for a duration of 7 days. Following acclimatization, the hamsters were randomly grouped and housed in separate cages, receiving food twice daily with unrestricted access to water. The fur surrounding the sebaceous plaque areas of the LVG hamsters was removed using a hair removal cream and a hair removal knife. The maximum transverse and longitudinal diameters of the sebaceous plaque areas were measured with Vernier calipers, and the area of the sebaceous plaque areas was calculated and recorded. In this study, five groups were established: a blank control group (deionized water), a positive control group (adapalene gel), a high-dose group (500 mg/mL LG-EPs), a medium-dose group (300 mg/mL LG-EPs), and a low-dose group (100 mg/mL LG-EPs). The topical doses of LG-EPs were selected according to the application amount of the positive control gel, the surface area of the sebaceous plaque areas, preliminary formulation feasibility, and local tolerance observations. The high dose of 500 mg/mL was set as the upper feasible concentration that could be uniformly applied without obvious precipitation or local irritation, whereas 300 mg/mL and 100 mg/mL were used as medium and low doses to establish a concentration gradient. In each group, 200 µL of LG-EPs was applied twice daily for 28 d. Body weights, sebaceous plaque areas, and images of the sebaceous plaque areas were recorded weekly. New hair around the sebaceous plaque areas was promptly removed. After 28 d of application and administration and following a 12-h fasting period without food and water, the LVG golden hamsters were euthanized via cervical dislocation. Subsequently, 2 cm×2 cm patches of sebaceous gland tissue were excised using forceps and a scalpel for further experimentation.

### HE staining and Oil Red O staining analysis

### HE staining analysis

Paraffin-embedded sections were deparaffinized in water and stained with hematoxylin for 3–5 min, followed by rinsing with tap water. Differentiation was performed using a differentiation solution, after which the sections were washed again with tap water and blued with a bluing reagent before being rinsed under running water. The samples were then dehydrated by sequential immersion in 85% and 95% ethanol (5 min each) and subsequently counterstained with eosin for 5 min. For clearing, the sections were successively immersed in absolute ethanol I, II, and III (5 min each), followed by xylene I and II for 5 min each. Finally, the slides were mounted with neutral gum, and microscopic examination was performed, with images captured for analysis.

### Oil Red O staining analysis

The frozen sections were rewarmed, dried, and fixed in fixative solution for 15 min. After being rinsed with tap water and dried, the sections were stained with Oil Red O solution for 8–10 min under light-protected conditions. The sections were then briefly air-exposed for 3 s and sequentially differentiated in two vats of 60% isopropanol for 3 s and 5 s, respectively. The samples were subsequently washed twice in pure water for 10 s each. After a brief exposure to air for 3 s, the sections were counterstained with hematoxylin for 3–5 min, followed by washing in three vats of pure water for 5 s, 10 s, and 30 s. Differentiation was performed using 60% alcohol for 2–8 s, and the sections were then rinsed in two vats of distilled water for 10 s each. After a brief immersion in blue solution (1 s), the sections were washed again in two vats of tap water for 5 s and 10 s. Finally, the slices were mounted with glycerol gelatin, and the staining results were observed under a microscope. Images were captured and analyzed.

###  Effect of LG-EPs on the contents of TG and FFAs in the tissues of golden hamsters

The triglyceride and protein contents in sebaceous glandular plaque tissue were determined using a TG assay kit and a BCA protein content assay kit from Biorigin Inc. (Beijing). A blank group, a standard group, and a sample group were established, and the specific experimental procedures were conducted according to the instruction manual. The absorbance value at 520 nm was measured to calculate the triglyceride content in the sebaceous glandular plaque tissue.

The FFA content of the sebaceous gland tissues from LVG hamsters was determined using a Solarbio free fatty acid content detection kit. Additionally, the protein content of the tissues was assessed using a BCA protein content detection kit from Biorigin Inc. (Beijing). The study included a control group, an assay group, a blank group, and a standard group, with experiments conducted according to the operating procedures outlined in the instruction manual. The absorbance was measured at 550 nm, and the content of free fatty acids in the sebaceous gland tissues was calculated on the basis of the standard curve.

### qRT‒PCR

The sequences of primers used in this study were designed using the NCBI platform. Total RNA extraction and cDNA synthesis were performed using TRIzol reagent and a reverse transcription kit according to the manufacturer’s instructions. Subsequent quantitative PCR analysis was conducted using the Fast Super EvaGreen^®^ qPCR Master Mix kit. The composition of the reverse transcription reaction system is shown in Table 5. The primer sequences used for cellular experiments and animal experiments are listed in Tables 6 and 7, respectively. The qRT–PCR reaction system used in this study is presented in Table 8.

**Table 5:** Reverse transcription system

Reagent	Volume (µL)
Total RNA	2.0
Anchored Oligo(Dt)18 Primer	1.0
2×ES Reaction Mix	10.0
EasyScript RT/RI Enzyme Mix	1.0
Gdna Remover	1.0
Rnase-free Water	5.0

**Table 6:** Primer sequences for cellular experiments

Gene	Direction	Primer (5’-3’)
IL-6	F	TTCTCCACAAGCGCCTTC
	R	AGAGGTGAGTGGCTGTCTGT
IL-8	F	GGAGAAGTTTTTGAAGAGGGCTG
	R	ACAGACCCACACAATACATGAAG
IL-1β	F	AACCTCTTCGAGGCACAAGG
	R	GTCCTGGAAGGAGCACTTCAT
TNF-α	F	TCTCCTTCCTGATCGTGGCA
	R	CAGCTTGAGGGTTTGCTACAAC
AQP3	F	CTTCTTTGACCAGGACCGGC
	R	GGGCCAGCTTCACATTCTCT
FLG	F	TGAGGCATACCCAGAGGACT
	R	CTGTATCGCGGTGAGAGGAT
LOR	F	CTTCCTGGTGCTTTGGGCTC
	R	CTGGGGGATCTATTTGGACGG
Caspase-14	F	ATTCCACGGTAGAGGGATACA
	R	TCAGGGTTCGTTTTCCTTGCT
PI3K	F	TCCCTTCGATAAGAGTCGAGG
	R	GCAGTCTTGTCGCAAAGTCC
AKT	F	AAGTCATCGTGGCCAAGGAC
	R	ACAGGTGGAAGAACAGCTCG
mTOR	F	CTTAGAGGACAGCGGGGAAG
	R	TCCTTTAATATTCGCGCGGC
Beta-actin	F	TGGCACCCAGCACAATGAA
	R	CTAAGTCATAGTCCGCCTAGAAGCA

**Table 7:** Animal primer sequences

Gene	Direction	Primer (5’-3’)
SREBP-1	F	ACCGACATCCAAGACATGCT
	R	CAAGCTCTCAGGAGAGTTGGC
FAS	F	GGTTCGTGAGGAGCCAGAG
	R	CCACCTAAGCCAGTGATGATG
ACC1	F	GCGTCATCTTGAACCTCCTCTGTC
	R	TCTTGATTGGCACTGGCTTGTCTG
GPER	F	CTCTGAACCGCTTCTGCCATG
	R	ACTGCTGAACCTGACCTCTGAC
ER-β	F	CCATAGACAAGAACCGGCGTAAGAG
	R	CCCACACTTGACCATCCCAACTTC
AR	F	TGCTCCACCGACATTAAAGACATCC
	R	GCTGCTGTTGCTGTTGCTGTTG
PI3K	F	TCCCTACCAAAGGCCAGGT
	R	CTTTGGTGGAAGAGCTTGAGGT
AKT	F	TAGCCATTGTGAAGGAGGGC
	R	CTTCATCAGCTGGCATTGTGC
mTOR	F	TCTGCAGGCCAGTCAGTAGAA
	R	AGAAATCCCGGCCAGTAAGC
AMPK	F	TCGGCAAAGTGAAGATTGGA
	R	TGGAGTGCTGATCACTTGGTAG
Beta-actin	F	GTACCACTGGCATTGTGATGGACTC
	R	GACGCTCGGTCAGGATCTTCATG

**Table 8:** PCR system

Reagent	Volume (µL)
Template	1.5
Forward Primer (10 µM)	0.4
Reverse Primer (10 µM)	0.4
2×TransStart^®^ Top Green qPCR SuperMix	10.0
Passive Reference Dye (50×)	0.4
Nuclease-free Water	7.3

### Data analysis

All experiments were conducted in triplicate. Data analysis and visualization were performed using GraphPad Prism 8, while mechanistic diagrams were created using Adobe Illustrator 2022. The significance of differences between groups was assessed using a t test and conventional one-way analysis of variance (ANOVA), with IC50 calculations performed using IBM Statistics 23. A p value of less than 0.05 was considered to indicate a significant difference between the groups.

## Results

### LG-EP molecular weight determination

The molecular weight of the LG-EPs was primarily between 1.0 × 10^4^ and 2.0 × 10^4^ g/mol. Molecules with molecular weights ranging from 1.0 × 10^4^ to 1.5 × 10^4^ g/mol accounted for approximately 40% of the total. The detailed molecular weight parameters are summarized in Table 9. The number-average molecular weight (Mn) of the LG-EPs was 1.576 × 10^4^ g/mol, the peak molecular weight (Mp) was 1.487 × 10^4^ g/mol, and the weight-average molecular weight (Mw) was 1.607 × 10^4^ g/mol. The polydispersity coefficient (PD) was 1.020.

**Table 9:** LG-EP molecular weight determination

LG-EP	
Molar mass moment (g/mol)	
Mn	1.576 × 10^4^ (± 1.584%)
Mp	1.487 × 10^4^ (± 1.531%)
Mw	1.607 × 10^4^ (± 1.605%)
Mz	1.659 × 10^4^ (± 3.610%)
Polydispersity	
Mw/Mn	1.020 (± 2.255%)
Mz/Mn	1.053 (± 3.943%)

### Peptide sequence identification

Bioactive peptides, also referred to as functional peptides, are specific protein fragments that positively influence various biological activities in living organisms and can significantly impact overall health. Owing to their unique biological properties, functional peptides hold great potential for applications in antibacterial, antiviral, anticancer, antioxidant, and immunomodulatory therapies (Xu Zhang et al. 2022). Antimicrobial peptides, whose molecular weights range from 1 to 10 kDa, are cationic, hydrophobic, and amphiphilic(Liu et al. 2023). They can inhibit or kill both gram-negative and gram-positive bacteria by targeting the cell membranes or internal structures of microorganisms (Lundy et al. 2020). Most antioxidant-active peptides contain hydrophobic amino acids, such as valine (Val) and leucine (Leu), at the N-terminal end and include amino acids such as proline (Pro), cysteine (Cys), tyrosine (Tyr), histidine (His), and tryptophan (Trp) throughout their entire sequence (Zhu et al. 2022; Yongtao Zhang et al. 2023b). On the basis of these properties, four peptides with antimicrobial potential and twelve peptides with antioxidant potential were selected, and their sequences and characteristics are listed in Tables 10 and 11, respectively.

**Table 10:** Peptides with antimicrobial potential

Name	Peptide	Mass	m/z
LG-EP-A	V.HASTNVNAADTTTTSV.S	1588.7329	795.3628
LG-EP-B	G.ESTLLMGAP.E	917.4528	459.7241
LG-EP-C	D.PNVASELFTSQ.A	1191.5771	596.7946
LG-EP-D	A.SATLSGSASREYNGQ.A	1526.696	764.3487

**Table 11:** Peptides with antioxidant potential

Name	Peptide	Mass	m/z
LG-EP-1	D.PVTGEIKY.N	905.4858	453.752
LG-EP-2	D.PEQQDVIRKL.M	1224.6826	409.2341
LG-EP-3	A.KETSYANFDATI.E	1358.6354	680.3228
LG-EP-4	N.DLTDENLYAYVTDA.D	1601.7097	801.8726
LG-EP-5	T.TPGVDPTDPKYKDM.F	1562.7286	782.3606
LG-EP-6	G.KDTFAGFTP.F	982.476	492.2346
LG-EP-7	N.SLDAGSSRIR.I	1060.5625	531.2825
LG-EP-8	D.SFTEYDAP.V	928.3814	465.2012
LG-EP-9	P.Q(+ 42.01)ALTDVTISSP.D	1172.5925	587.2974
LG-EP-10	D.PEGPKYPTDSA.N	1160.5349	581.2653
LG-EP-11	L.ITNSATKYGHVSWQN.M	1704.822	853.4139
LG-EP-12	E.KDGLNEVP.E	870.4446	436.2289

### Safety determination

**Fig. 2:**
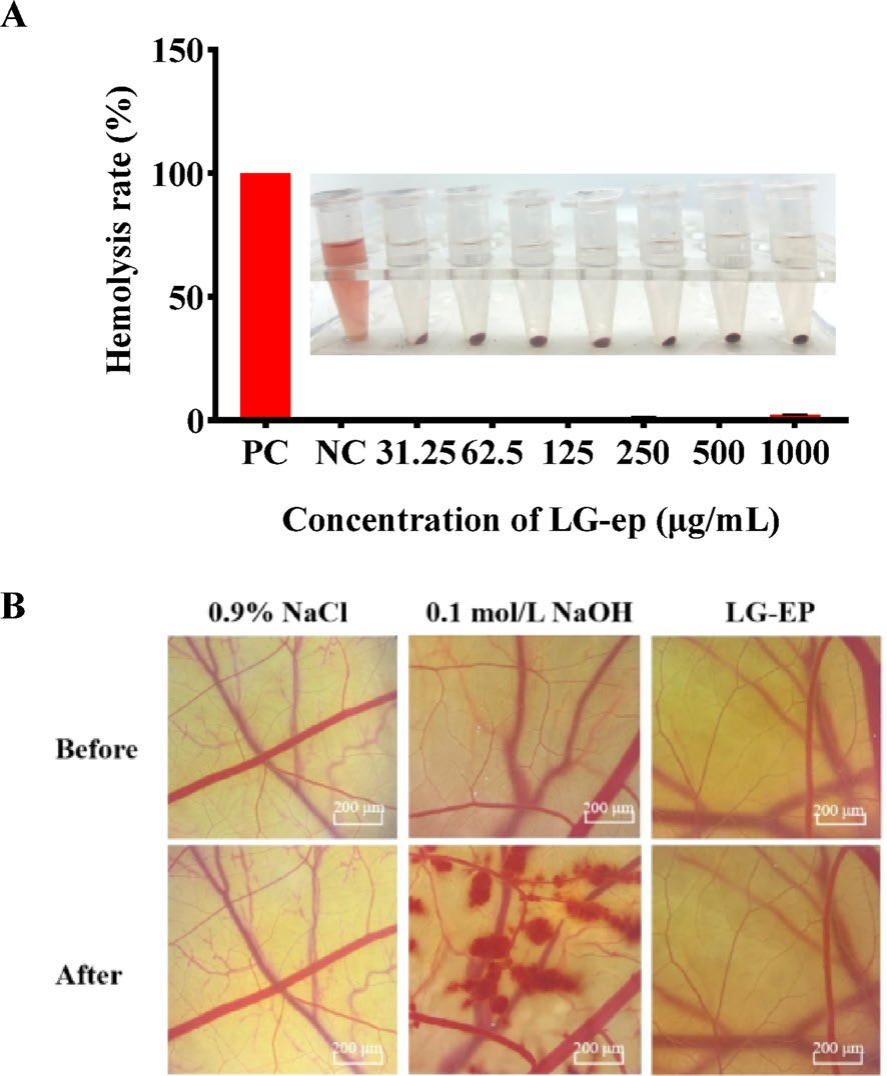
Safety determination of LG-EPs. (**A**) Effect of LG-EPs on erythrocyte hemolysis. (**B**) Effects of LG-EPs on the vasculature of chick embryos

The erythrocyte hemolysis (RBC) test is commonly used to evaluate the safety of samples by measuring the leakage of hemoglobin from erythrocytes. The results of the rabbit erythrocyte hemolysis assay are shown in Fig. 2A. The effects of different concentrations of LG-EPs on the hemolysis of rabbit erythrocytes were consistently low, indicating that LG-EPs exhibited negligible hemolytic activity and can therefore be considered safe. In addition, the safety of LG-EPs was further evaluated using the chick embryo chorioallantoic membrane (HET-CAM) eye irritation test. As shown in Fig. 2B, the positive control (0.1 mol/L NaOH) produced a strong stimulatory effect on blood vessels, resulting in an irritation score of 12. In contrast, LG-EPs did not cause visible damage to the blood vessels on the chorioallantoic membrane. The detailed irritation scores and grading results are summarized in Table 12. No hemorrhage, coagulation, or vascular lysis was observed in the LG-EP group, indicating that LG-EPs exhibit excellent safety characteristics.

**Table 12:** LG‒EPs safety test

Sample	Bleeding type	Rating/Grading						ES/point
		1	2	3	4	5	6	
Negative control (0.9% NaCl)	Hemorrhage	0	0	0	0	0	0	0
	Coagulation	0	0	0	0	0	0	
	Vascular lysis	0	0	0	0	0	0	
Positive control (0.1 mol/L NaOH)	Hemorrhage	2	2	2	2	2	2	12
	Coagulation	2	1	1	1	2	1	
	Vascular lysis	1	1	1	1	1	1	
LG-EPs	Hemorrhage	0	0	0	0	0	0	0
	Coagulation	0	0	0	0	0	0	
	Vascular lysis	0	0	0	0	0	0	

### Antibacterial activity of LG-EPs against acne-associated bacteria

#### MIC and MBC of LG-EPs against *C. acnes* and *S. aureus*

The minimum inhibitory concentration (MIC) and minimum bactericidal concentration (MBC) are important indices for evaluating the antibacterial activity of a sample. In general, lower MIC and MBC values indicate stronger antibacterial activity. The antibacterial effects of LG-EPs against two common acne-associated bacteria, *C. acnes* and *S. aureus*, were evaluated by coculture with gradient concentrations, and the results are summarized in Table 13. LG-EPs inhibited the growth of both bacterial species. For *C. acnes*, the MIC and MBC values were both 600 µg/mL. For *S. aureus*, the MIC was 700 µg/mL and the MBC was 1 mg/mL.

**Table 13:** Minimum inhibitory concentration and minimum bactericidal concentration of LG-EPs against *Cutibacterium acnes* and *Staphylococcus aureus*

	C.acnes	S. aureus
MIC	600 µg/mL	700 µg/mL
MBC	600 µg/mL	1 mg/mL

#### LG-EPs inhibit the growth of *C. acnes* and *S. aureus*

**Fig. 3:**
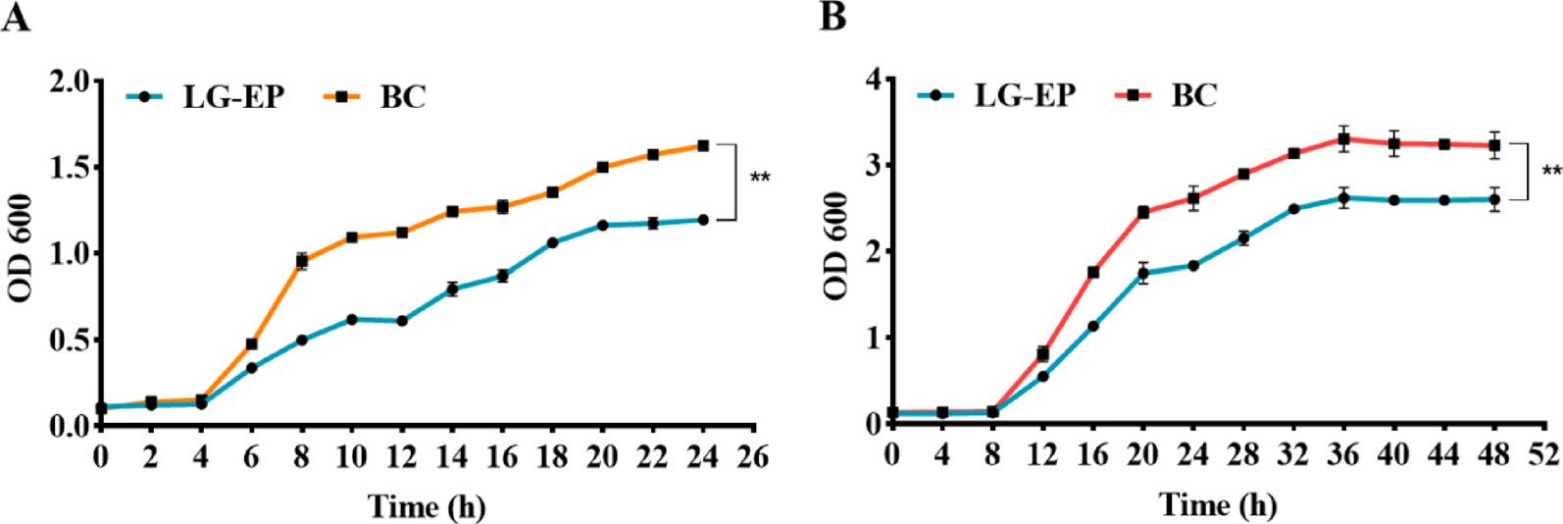
Effect of MIC concentration of LG-EPs on the growth curves of *S. aureus* and *C. acnes*. (**A**) Effect of 700 µg/mL LG-EPs on the growth curve of *S. aureus*; (**B**) Effect of 600 µg/mL LG-EPs on the growth curve of *C. acnes*. **p < 0.01, compared with the blank control group

Growth-curve analysis helps to better characterize bacterial growth behavior. The effects of LG-EPs at MIC concentrations on the growth curves of *C. acnes* and *S. aureus* are shown in Fig. 3. In the blank control group, both *C. acnes* and *S. aureus* exhibited normal growth patterns. *S. aureus* entered the logarithmic growth phase at approximately 4 h and reached the stationary phase after 22 h, whereas *C. acnes* entered the logarithmic phase at approximately 8 h and reached the stationary phase after 36 h. Compared with the blank control group, LG-EPs at the MIC significantly inhibited the growth of both bacteria. Specifically, the LG-EP-treated groups exhibited lower OD600 values at the same time points and slower proliferation rates, indicating that LG-EPs exerted a marked inhibitory effect on bacterial growth.

#### LG-EPs inhibit biofilm adhesion of *C. acnes* and *S. aureus*

**Fig. 4:**
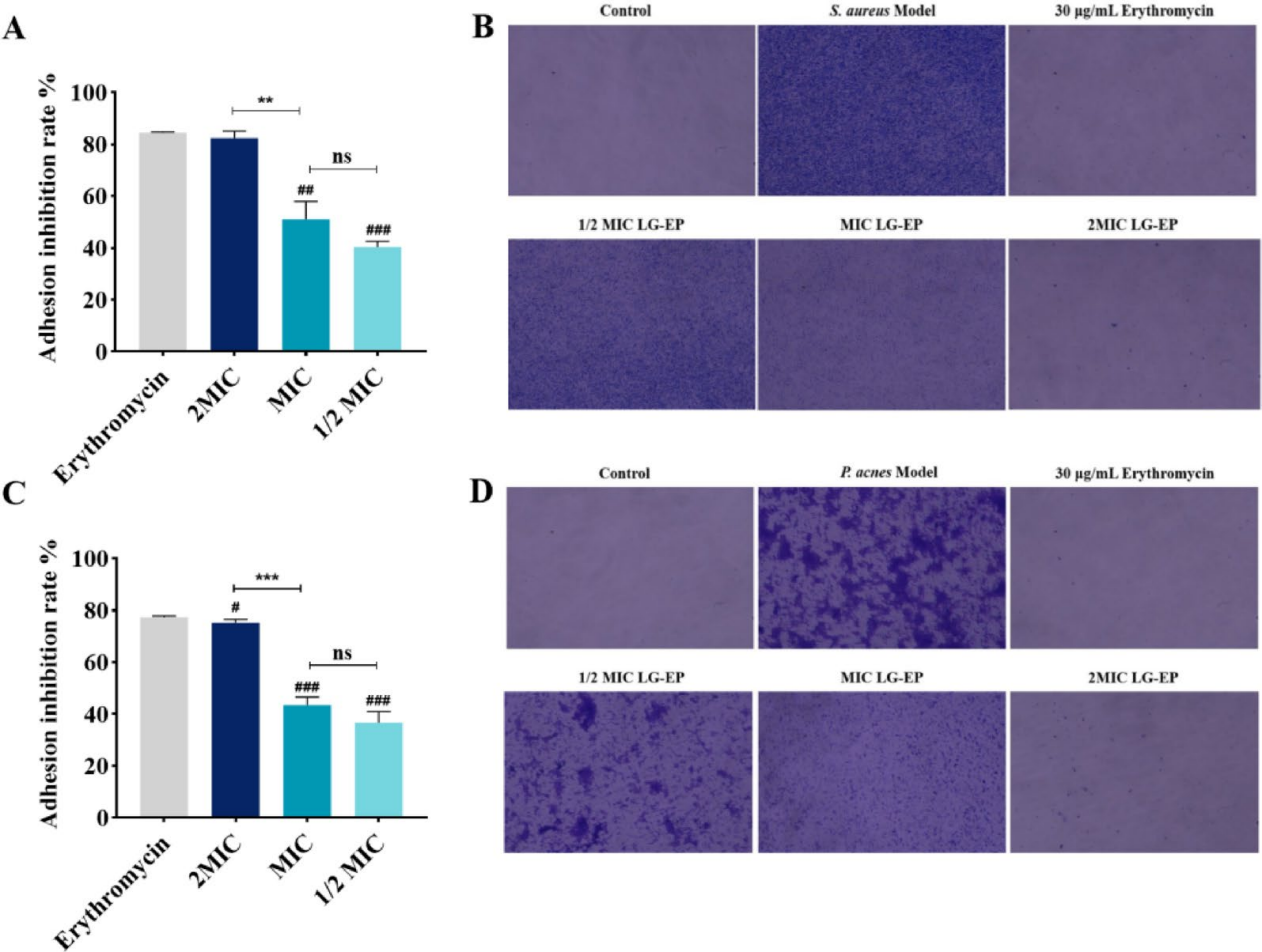
Effects of LG-EPs on biofilm adhesion of *S. aureus* and *C. acnes*, two acne pathogens. (**A**) & (**B**) Effect of 700 µg/mL LG-EP on biofilm adhesion of *S. aureus*; (**C**) & (**D**) Effect of 600 µg/mL LG-EP on biofilm adhesion of *C. acnes*. #*p* < 0.05, ##*p* < 0.01, ###*p* < 0.001, compared with 30 µg/mL erythromycin; ^ns^*p*>0.05, **p < 0.01, ***p < 0.001, compared with MIC concentration of LG-EP

Using erythromycin (30 µg/mL) as a positive control, the effects of LG-EPs on biofilm adhesion of *C. acnes* and *S. aureus* were determined by crystal violet staining, and the results are shown in Fig. 4. LG-EPs at 1/2 MIC, MIC, and 2 MIC significantly inhibited the biofilm adhesion of both bacteria. At 2 MIC, the inhibitory effects of LG-EPs on biofilm adhesion of *C. acnes* and *S. aureus* were not significantly different from those of erythromycin. At 1/2 MIC, the inhibition rates of LG-EPs against biofilm adhesion were 36.68% for *C. acnes* and 40.36% for *S. aureus*. Overall, the inhibitory effect of LG-EPs on bacterial biofilm adhesion increased with increasing concentration.

### In vitro anti-inflammatory and antioxidant activity assays

**Fig. 5:**
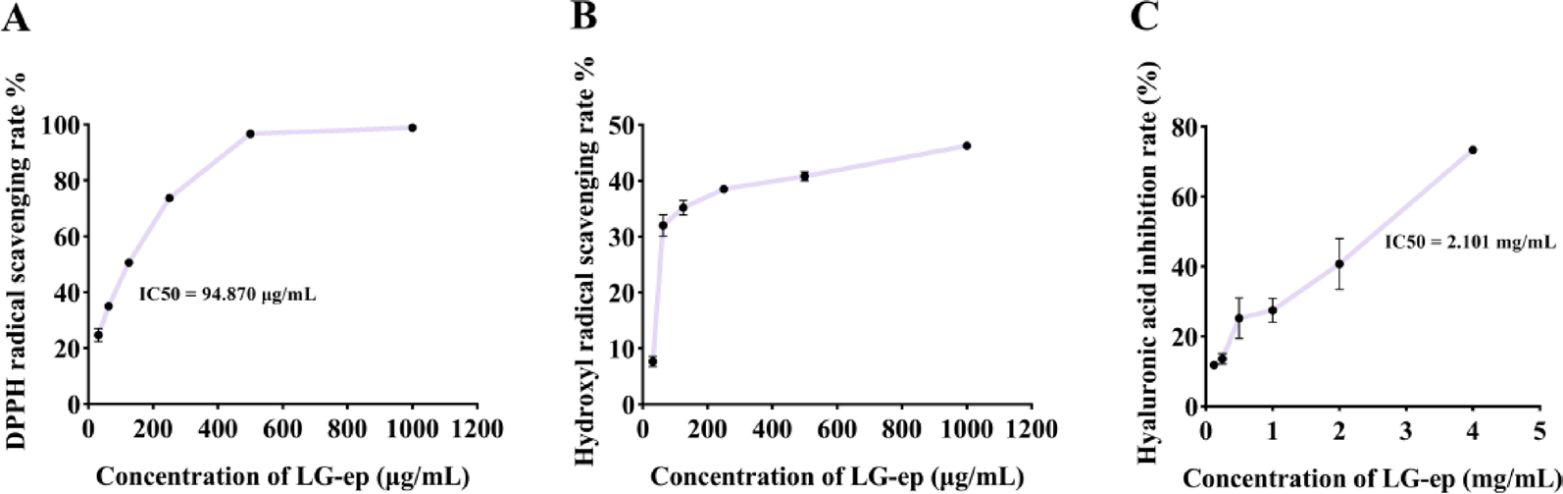
Determination of the anti-inflammatory capacity of LG-EPs for in vitro antioxidant activity. (**A**) Determination of the scavenging capacity of LG-EPs for DPPH free radicals. (**B**) Determination of the hydroxyl radical scavenging capacity of LG-EPs. (**C**) Determination of the ability of LG-EPs to inhibit hyaluronidase activity

The in vitro antioxidant capacity of LG-EPs was initially evaluated using DPPH and hydroxyl radical scavenging assays. LG-EPs displayed notable scavenging activity against both radicals. Specifically, 94.87 µg/mL LG-EPs achieved 50% scavenging of DPPH radicals (Fig. 5A), while 250 µg/mL resulted in 38.51% hydroxyl radical scavenging (Fig. 5B). Furthermore, inhibition of hyaluronidase activity and the consequent reduction in small-molecule hyaluronic acid are considered beneficial for alleviating skin inflammation (Chaiyana et al. 2020). In the hyaluronidase inhibition assay, 2.10 mg/mL LG-EPs inhibited enzyme activity by 50% (Fig. 5C). Overall, these findings demonstrate that LG-EPs possess significant in vitro antioxidant and anti-inflammatory activities, highlighting their potential as a cosmetic ingredient with dual functional benefits.

### Modeling of LPS injury and determination of cell viability and ROS content

**Fig. 6:**
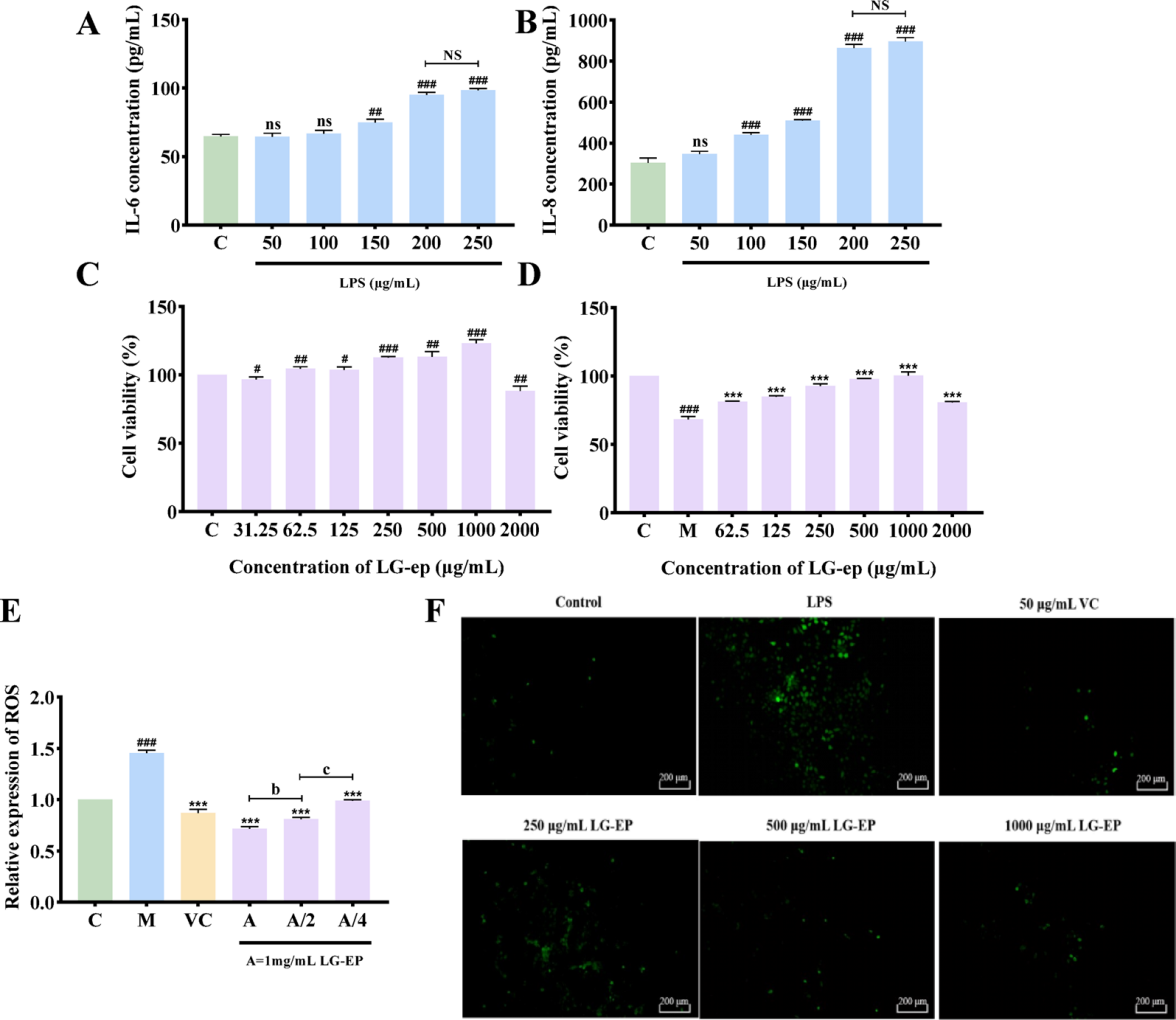
Modeling of LPS-induced inflammatory injury, investigation of the effect of LG-EPs on cell viability, and assessment of the impact of LG-EPs on LPS-induced oxidative stress injury. (**A**) Effect of different concentrations of LPS on the IL-6 concentration in HaCaT cells after treatment with LPS. (**B**) Effect of different concentrations of LPS on the IL-8 concentration in HaCaT cells after treatment. (**C**) Effect of LG-EPs on HaCaT cell viability. (**D**) Effect of LG-EPs on the impairment of HaCaT cell viability caused by LPS. (**E**) Effect of LG-EPs on the ROS concentration. (**F**) Effect of LG-EPs on the fluorescence content of ROS. (^ns^*p*>0.05, ^#^*p*<0.05, ^##^*p*<0.01, ^###^*p*<0.001 vs. the control group; ^***^p<0.001 vs. the model group; ^NS^*p*>0.05, ^a^*p*<0.05, ^b^*p*<0.01, ^c^*p*<0.001 vs. the A/2 LG-EPs)

Lipopolysaccharide (LPS) is typically used to establish inflammatory injury models. In this study, LPS was applied to HaCaT cells to induce inflammatory damage. Interleukin-6 (IL-6) and interleukin-8 (IL-8) were selected as indicators to evaluate model establishment (Vilotić et al. 2022). As shown in Fig. 6A and B, the expression of both IL-6 and IL-8 increased with increasing LPS concentration, but the difference did not significantly differ between the groups treated with 200 µg/mL and 250 µg/mL LPS. Therefore, treatment with 200 µg/mL LPS for 24 h was chosen as the optimal condition for constructing the inflammatory model.

The influence of LG-EPs on HaCaT cell viability was assessed using a CCK-8 assay. As illustrated in Fig. 6C, concentrations less than 2000 µg/mL resulted in no cytotoxicity, and the viability was maintained above 80%. At 2000 µg/mL, a mild reduction in viability was detected, likely due to concentration-related toxicity. Treatment with 200 µg/mL LPS for 24 h markedly reduced cell survival, whereas treatment with 250 µg/mL, 500 µg/mL, and 1000 µg/mL LG-EPs significantly restored cell viability, indicating a protective effect against LPS-induced damage (Fig. 6D).

Oxidative stress and inflammation reinforce each other: oxidative stress triggers inflammatory responses, whereas inflammation prolongs and intensifies oxidative stress, resulting in a vicious cycle (Lin et al. 2019). As depicted in Fig. 6E and F, compared with control cells, HaCaT cells in the LPS-stimulated model group exhibited markedly stronger green fluorescence and elevated ROS levels, indicating oxidative stress-induced cellular injury. Treatment with LG-EPs at different concentrations reduced the fluorescence intensity, decreased the ROS content, and alleviated oxidative damage. Among them, 1000 µg/mL LG-EPs produced the greatest reduction in ROS, effectively mitigating oxidative stress and surpassing the clearance effect observed with 50 µg/mL vitamin C (VC).

### LG-EPs alleviates inflammatory injury and barrier damage caused by LPS

IL-6, IL-8, IL-1β, and TNF-α are key proinflammatory cytokines that play crucial roles in LPS-induced inflammation in the skin. TNF-α and IL-1β serve as initiators of the inflammatory response, while IL-6 further amplifies inflammatory signals, and IL-8 exacerbates the localized response by recruiting inflammatory cells (Erdinest et al. 2014). The synergistic action of these factors leads to typical inflammatory symptoms, such as skin redness, swelling, heat, and pain (Veltri et al. 2021). Aquaporin 3 (AQP3), filaggrin (FLG), loricrin (LOR), and caspase-14 are essential molecules involved in skin barrier function (Rui Wang et al. 2023). They are very important for keeping the skin moist, sustaining the well-being of the stratum corneum, and preserving overall barrier function (Kim et al. 2022). The expression or activity of these molecules can be impaired under conditions of LPS-induced skin barrier damage, resulting in fragile barrier integrity. An important approach may comprise the restoration of the expression or activity of these important molecules as a method of mending the skin barrier.

**Fig. 7:**
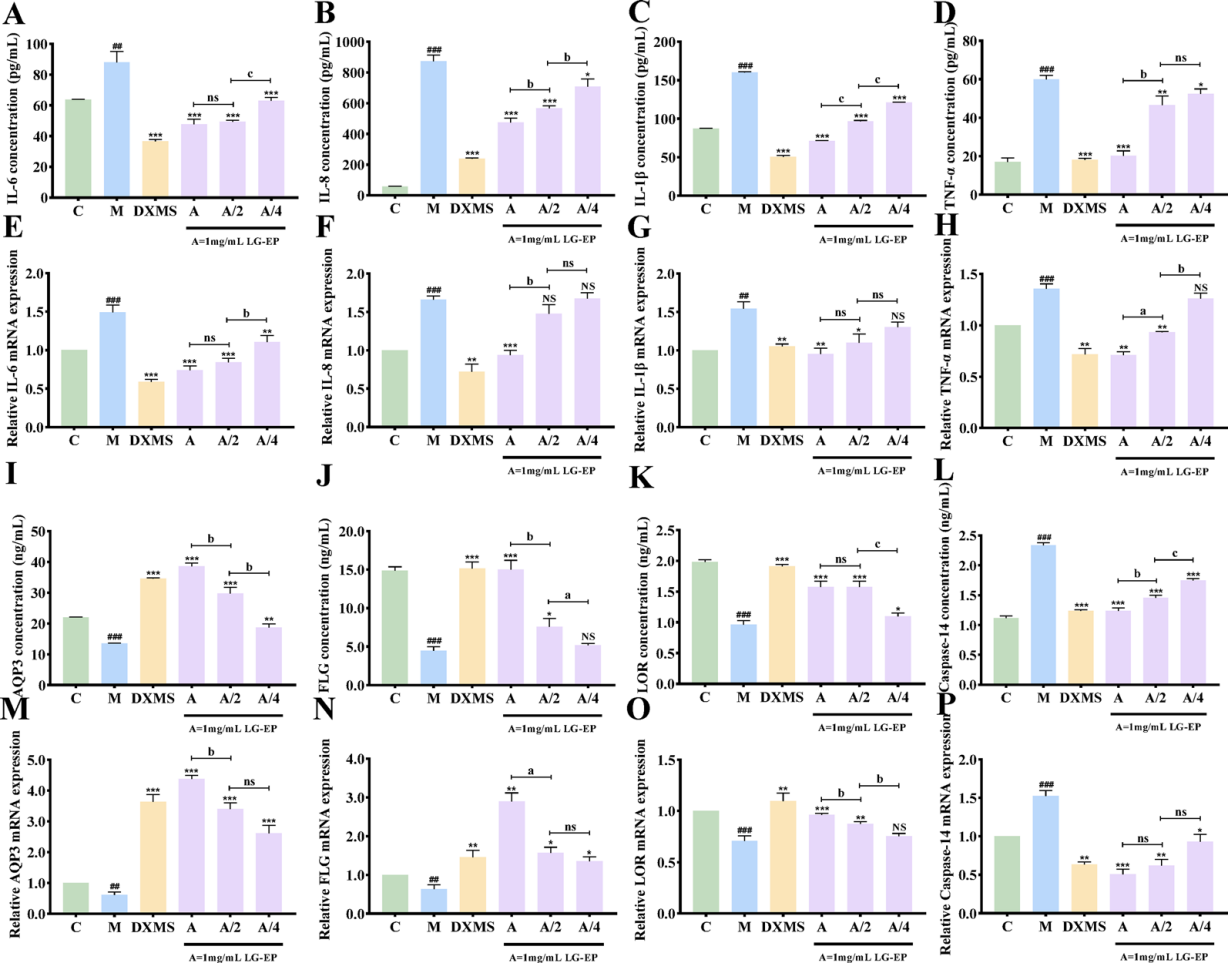
Effects of LG-EPs on the levels and transcription of inflammation-related and barrier-related factors in LPS-injured HaCaT cells.**A**& **E**. Effects of LG-EPs on the IL-6 content and transcript levels. **B**& **F**. Effects of LG-EPs on the IL-8 content and transcript levels.**C**& **G**. Effects of LG-EPs on the IL-1β content and transcript levels. **D** & **H**. Effects of LG-EPs on the TNF-α content and transcript levels. **I** & **M**. Effects of LG-EPs on the AQP3 content and transcript levels. **J** & **N**. Effects of LG-EPs on the FLG content and transcript levels. **K** & **O**. Effects of LG-EPs on the LOR content and transcript levels. **L** & **P**. Effects of LG-EPs on the caspase-14 content and transcript levels. (^##^*p*<0.01, ^###^*p*<0.001 vs. the control group; ^NS^*p*>0.05, ^*^p<0.05, ^**^p<0.01, ^***^p<0.001 vs. the model group; ^ns^*p*>0.05, ^a^*p*<0.05, ^b^*p*<0.01, ^c^*p*<0.001 vs. the A/2 LG-EPs; DXMS, dexamethasone)

With respect to the inflammatory response, as shown in Fig. 7A-H, the protein content and mRNA transcripts of the four inflammatory factors (IL-6, IL-8, IL-1, and TNF-α) significantly increased after LPS stimulation compared with those in the control group. However, this trend was reversed when the cells were treated with different concentrations of LG-EPs. Unlike those in the model group, the levels of the inflammatory markers decreased in a dose-dependent manner in response to LG-EP intervention (Fig. 7A–H). Notably, compared with the positive control (dexamethasone, DXMS), the increased concentrations of LG-EPs resulted in a stronger inhibitory response to the effect of LPS on inflammation.

In terms of skin barrier function, as shown in Fig. 7I-P, the expression of barrier-related proteins was significantly disrupted by LPS stimulation. The expression of AQP3, FLG and LOR decreased considerably in the control group compared with that in the model group, and the level of caspase-14 expression increased significantly, indicating impaired barrier function.

### Effect of LG-EPs on the PI3K/AKT/mTOR signaling pathway

The PI3K/AKT/mTOR pathway plays a crucial role in the inflammatory response of the skin and the development of acne (Lesiak et al. 2024). This pathway influences sebaceous gland function and the behavior of keratin-forming cells by regulating cell proliferation, differentiation, and metabolism (Jurca et al. 2023). Overactivation of the PI3K/AKT/mTOR signaling pathway leads to increased sebum secretion and abnormal follicular keratinization, resulting in follicular blockage and creating conditions conducive to the proliferation of *Cutibacterium acnes*, which subsequently triggers an inflammatory response (Tong et al. 2022). Furthermore, this pathway exacerbates local inflammation by activating inflammatory factors such as NF-κB, contributing to the characteristic pathological features of acne (Leyuan Wang et al. 2021). Therefore, inhibiting the overactivation of the PI3K/AKT/mTOR pathway may represent a novel strategy for acne treatment.

**Fig. 8:**
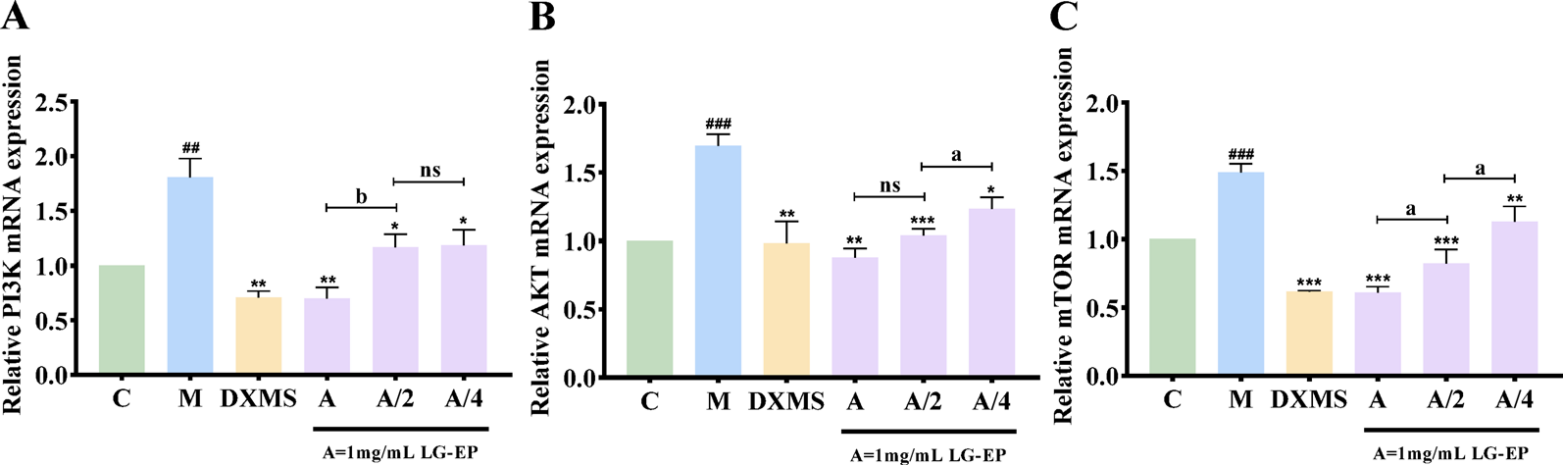
Effects of LG-EPs on the PI3K/AKT/mTOR signaling pathway. (**A**) Effect of LG-EPs on PI3K gene transcript levels. (**B**) Effect of LG-EPs on AKT gene transcript levels. (**C**) Effect of LG-EPs on mTOR gene transcript levels. (^##^*p*<0.01, ^###^*p*<0.001 vs. the control group; ^*^p<0.05, ^**^p<0.01, ^***^p<0.001 vs. the model group; ^ns^*p*>0.05, ^a^*p*<0.05, ^b^*p*<0.01 vs. the A/2 LG-EP; DXMS, dexamethasone)

As shown in Fig. 8A–C, compared with that in the control group, the relative mRNA expression of three key genes—PI3K, AKT, and mTOR—in the model group significantly increased following LPS-induced injury, indicating pathway hyperactivation. However, treatment with varying concentrations of LG-EPs effectively reversed this upregulation. Compared with those in the model group, the transcript levels of these three genes decreased to varying degrees in the LG-EP-treated groups (Fig. 8). Notably, the inhibitory effect of 1 mg/mL LG-EPs on the transcript levels of PI3K, AKT, and mTOR was comparable to that of the positive control (10 µg/mL dexamethasone), with no statistically significant difference observed (Fig. 8A–C). These results suggest that LG-EPs mitigate inflammation potentially by suppressing the PI3K/AKT/mTOR signaling cascade.

### Effect of LG-EPs on sebaceous plaque areas in golden hamsters

**Fig. 9:**
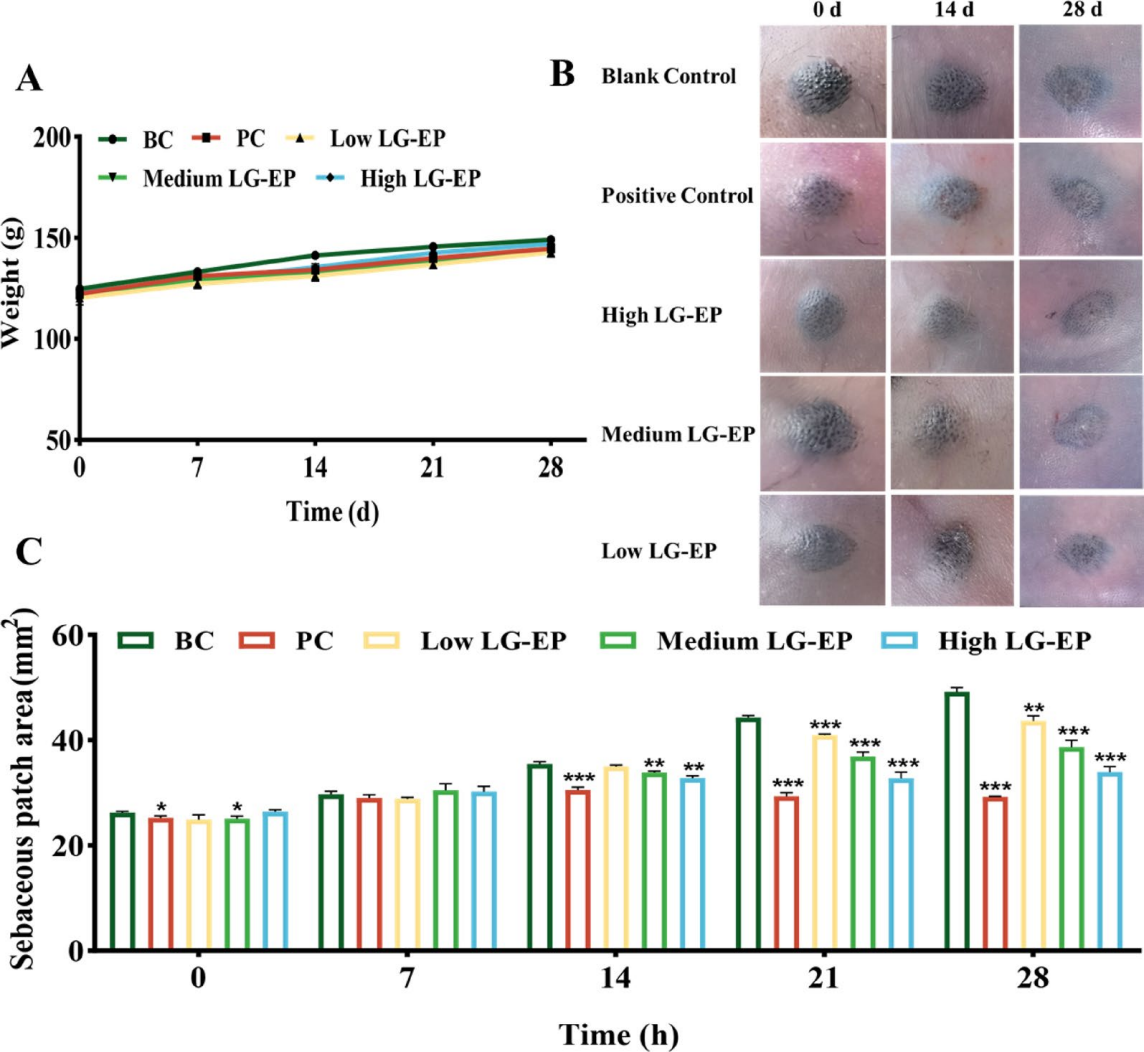
Effects of LG-EPs on body weight and sebaceous plaque areas in golden hamsters. (**A**) Effect of LG-EPs on the body weight of golden hamsters. (**B**) Effect of LG-EPs on the color and size of sebaceous plaque areas in golden hamsters. (**C**) Effect of LG-EPs on the size of the sebaceous plaque areas in golden hamsters. (^**^p<0.01, ^***^p<0.001 vs. the BC group)

The sebaceous plaque areas on the back of the golden hamster served as a natural model for investigating the effects of various samples on sebum secretion, eliminating the need for artificial modeling treatments. LG-EP samples at concentrations of 100, 300, and 500 mg/mL were applied to the hamsters. The positive control group was treated with adapalene gel, which was applied to the hamsters. As shown in Fig. 9A, the body weight did not significantly differ between the LG-EP-treated groups, the positive control group, and the blank control (BC) group throughout the experiment. These findings indicate that the topical application of LG-EPs does not affect the drinking or feeding behaviors or general health of the animals, suggesting a favorable safety profile.

With respect to the macroscopic changes, Fig. 9B provides representative images of sebaceous plaque areas. The size, color, and protrusion of the sebaceous plaque areas in the BC group did not vary much during the experimental period. Conversely, compared with the BC group, the positive control and medium- and high-dose LG-EP groups presented a significant reduction in spot pigmentation (Fig. 9B). The demarcation lines between the sebaceous plaque areas and the rest of the skin became vague, and the degree of spot protrusions was physically diminished.

Quantitative analysis of the spot area further confirmed these observations (Fig. 9C). Compared with no treatment, treatment with LG-EPs significantly reduced the area of sebaceous plaque areas, indicating an inhibitory effect on sebaceous gland activity.

### HE staining and Oil Red O staining analysis

**Fig. 10:**
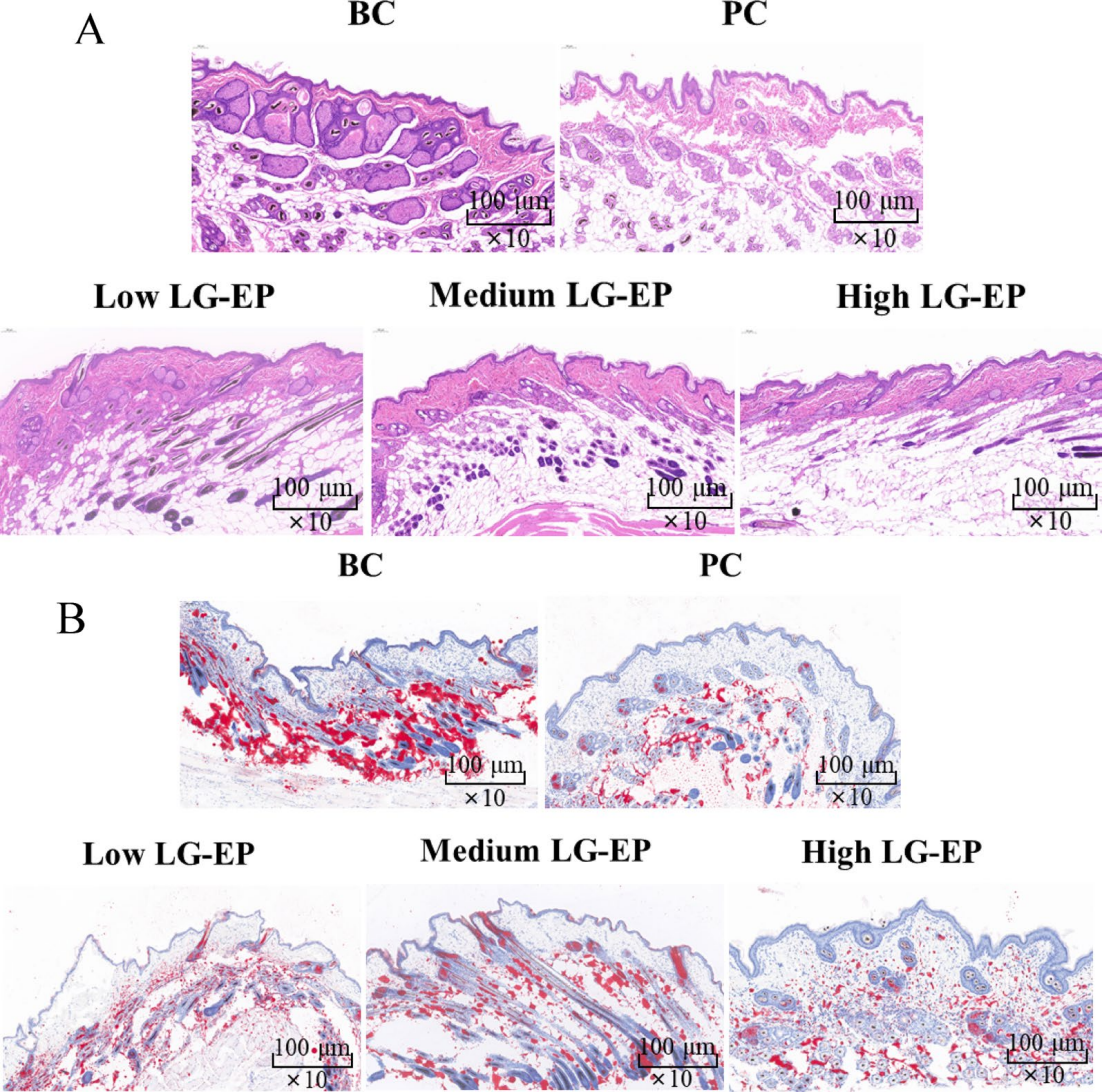
Effects of LG-EPs on HE staining and Oil Red O staining. (**A**) Effect of LG-EPs on H&E staining. (**B**) Effect of LG-EPs on Oil Red O staining

Skin histopathological changes were analyzed using H&E staining (Fig. 10A). In the blank control group (Group C), the sebaceous glands appeared lobulated, with larger, denser, and more tightly arranged glands, accompanied by numerous surrounding glandular cells. In contrast, the positive control group (Group PC) exhibited atrophied sebaceous glands that were smaller in size and more loosely arranged than those in Group C were. Following the application of high, medium, and low doses of LG-EPs, the sebaceous glandular vesicles in the sample group were significantly reduced compared with those in Group C. The glands appeared shrunken, smaller, and loosely arranged, taking on a pike-like shape. The alterations in the microstructure of the sebaceous glands were most pronounced in the high-dose sample group.

0O0il Red O is an azo dye known for its strong lipid solubility and ability to stain lipids, allowing for it to bind with triglycerides to form small lipid droplets (Kraus et al. 2016). When Oil Red O is applied to frozen tissue sections, it dissolves in the lipids within the tissues, resulting in an orange‒red coloration (Du et al. 2023). As illustrated in Fig. 10B, in the blank control group, numerous clustered red lipid droplets were observed within the cytoplasm of sebaceous gland cells in the sebaceous gland tissue of the golden hamster. These droplets fused into the lamellae, indicating exuberant lipid secretion. In the positive control group, the accumulation of orange‒red lipid droplets around the glandular follicles was significantly reduced compared with that in Group C. Following the application of high, medium, and low doses of LG-EPs, a decreasing trend in sebum secretion was noted across all sample groups compared with Group C. The inhibitory effect on lipid secretion was most pronounced in the high-dose sample group.

In summary, LG-EPs can inhibit sebum secretion by reducing the size of sebaceous gland lobes in sebaceous gland tissues and decreasing lipid accumulation in sebaceous cells.

### Effect of LG-EPs on the contents of TG and FFAs in the tissues of golden hamsters

**Fig. 11:**
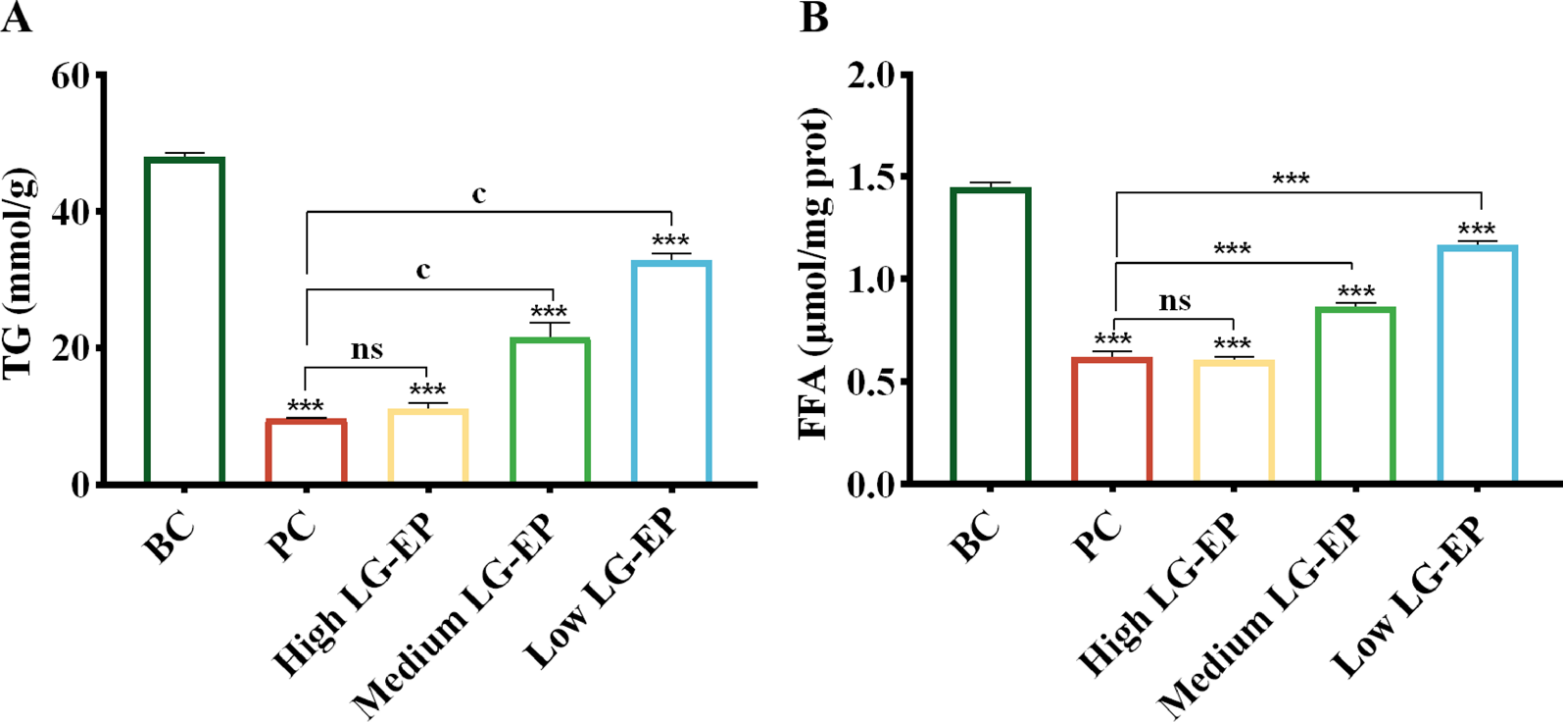
Effects of LG-EPs on the TG and FFA contents of sebaceous plaque areas in golden hamster. (**A**) Effect of LG-EPs on the TG content. (**B**) Effect of LG-EPs on the FFA content. (^***^p<0.001 vs. BC group; ^ns^*p*>0.05, ^c^*p*<0.001 vs. PC group)

The levels of triglycerides (TG) and free fatty acids (FFA) are important indicators for evaluating sebum production, as they are the primary components of sebum, accounting for 57.5% of its total composition (Jong et al. 1998). As illustrated in Fig. 11A and B, compared with those in the blank control group, the TG and FFA contents in the sample group significantly decreased. Furthermore, the inhibitory effect of LG-EPs at high doses on the synthesis of TG and FFAs did not significantly differ from that of the positive control group.

### Effect of LG-EPs on sebum secretion in the skin tissue of golden hamsters

Sterol regulatory element-binding protein-1 (SREBP-1) is essential for steroid synthesis, whereas fatty acid synthase (FAS) and acetyl coenzyme A carboxylase 1 (ACC1) are the two primary downstream targets of SREBP-1, both of which play significant roles in regulating sebum production (Er-Min Wang et al. 2018). LG-EPs inhibit sebum production by suppressing changes in the expression of adipogenic genes associated with SREBP-1, FAS, and ACC1. SREBP-1 activation is regulated by the PI3K/AKT/mTOR pathway, which is triggered by specific receptors activated by various factors, including cytokines (Farrash et al. 2024). This pathway regulates the protein expression of lipid synthases by modulating SREBP-1 activity. Additionally, AMP-activated protein kinase (AMPK) signaling influences adipogenesis in human sebocytes. AMPK serves as a major negative regulator of mTOR, which is crucial for lipid metabolism, and it inhibits the synthesis of key lipogenic enzymes in various tissues by preventing the formation of SREBP-1 (Li et al. 2025). Consequently, LG-EPs may inhibit sebum secretion by activating AMPK signaling, thereby reducing the expression of mTOR or directly inhibiting the activity of SREBP-1.

**Fig. 12:**
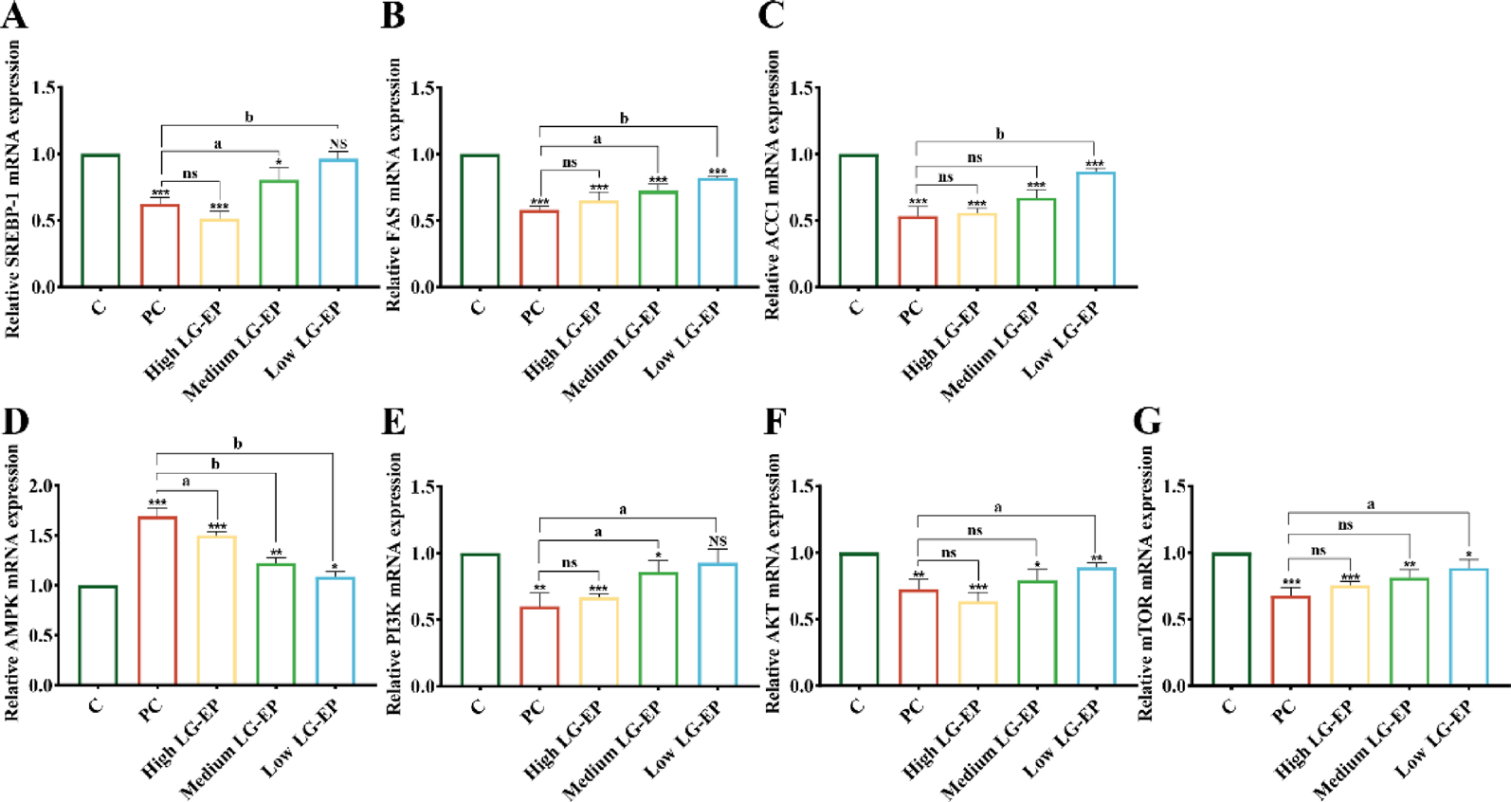
Effects of LG-EPs on genes closely related to sebum secretion at the sebaceous plaque areas of golden hamster and the underlying mechanism. (**A**) Effect of LG-EPs on SREBP-1 gene transcript levels. (**B**) Effect of LG-EPs on FAS gene transcript levels. (**C**) Effect of LG-EPs on ACC1 gene transcript levels. (**D**) Effect of LG-EPs on AMPK gene transcript levels. (**E**) Effect of LG-EPs on PI3K gene transcript levels. (**F**) Effect of LG-EPs on AKT gene transcript levels. (**G**) Effect of LG-EPs on mTOR gene transcript levels. (^NS^*p*>0.05, ^*^p<0.05, ^**^p<0.01, ^***^p<0.001 vs. the BC group; ^ns^*p*>0.05, ^a^*p*<0.05, ^b^*p*<0.01 vs. the PC group)

The experimental results in Fig. 12A–C revealed that different concentrations of LG-EPs could effectively inhibit the transcription of SREBP-1, FAS and ACC1 after acting on the sebaceous gland sites of golden hamster, and the higher the concentration of the samples was, the more obvious the inhibitory effect was. The inhibitory effect of the high-dose concentration of LG-EPs on the transcription level of the three genes did not significantly differ from that of the positive control group. The experimental results shown in Fig. 12D–G suggest that LG-EPs may inhibit sebum secretion by activating AMPK signaling, which in turn inhibits the expression of mTOR or directly inhibits the activation of the PI3K/AKT/mTOR signaling pathway.

## Discussion

Acne, a common chronic inflammatory skin disease, has become a significant global health concern. Statistics indicate that the prevalence of acne is as high as 85% among adolescents and 12–14% among adults, with a continuous upward trend observed in recent years (Dréno et al. 2020). The pathogenesis of acne is complex and involves four key pathological aspects: first, hyperactivity of the sebaceous glands leads to a substantial increase in sebum production, creating a favorable environment for microbial proliferation; second, abnormal keratinization of the follicular sebaceous gland ducts results in pore clogging; third, overproliferation of microorganisms such as *Cutibacterium acnes* (*C. acnes*) triggers inflammatory responses; and finally, abnormal activation of the immune system results in the release of inflammatory mediators and the formation of inflammatory lesions (Kokandi 2010). On the basis of the characteristics and extent of the lesions, acne can be divided into mild (mainly composed of pimples), moderate (inflammatory papules and pustules), and severe (nodules and cysts) (Fabbrocini et al. 2010). In the treatment of acne of different severities, modern medical treatments mainly combine topical (retinoids, benzoyl peroxide, and antibiotics), systemic (oral antibiotics and isotretinoin) and physical (photodynamic therapy and laser therapy) treatments (Frénard et al. 2021). Nevertheless, the pathogenesis of acne has still not been fully studied, and each patient is unique; thus, creating more tailored and successful treatment programs remains one of the primary interests of research and a challenge in dermatology.

In recent years, increasing attention has been paid to the use of probiotics and probiotic-derived metabolites in acne management. Zhang et al. reported that extracellular proteins from *Weizmannia coagulans* could reduce skin acne by inhibiting acne-associated pathogenic bacteria and regulating inflammation-related signaling pathways. *Lactobacillus gasseri* is widely recognized as a safe probiotic and has been extensively used in food and medical fields (Arakawa et al. 2015). This strain possesses multiple probiotic properties, including regulation of intestinal microbiota, enhancement of host immune function, and inhibition of pathogenic bacteria (Wu et al. 2023). In addition, *L. gasseri* has been reported to alleviate inflammatory responses, indicating its potential biological relevance in inflammation-related conditions (Sun et al. 2020). However, most existing studies on L. gasseri have focused mainly on viable bacterial cells or general probiotic effects, whereas systematic investigations of its extracellular proteins remain limited.

Compared with conventional probiotic or metabiotic approaches, LG-EPs represent a cell-free and protein-rich fraction, which may offer better stability, safety, and component controllability than live bacterial preparations or undefined postbiotic mixtures. The innovation of the present study lies in specifically identifying L. gasseri extracellular proteins as the research object and evaluating their anti-acne potential through an integrated strategy combining physicochemical characterization, antibacterial assays, cellular inflammatory models, and an in vivo sebaceous gland model. In the revised study, LG-EPs showed direct inhibitory effects against acne-associated bacteria, including *Cutibacterium acnes* and *Staphylococcus aureus*, as demonstrated by MIC, MBC, growth-curve, and biofilm adhesion assays. These newly added results extend the anti-acne relevance of LG-EPs beyond anti-inflammatory and sebum-suppressive effects. Taken together, our findings suggest that LG-EPs may exert multi-target anti-acne activity by inhibiting acne-associated bacterial growth and adhesion, suppressing inflammatory responses, improving barrier-related factors, and reducing sebum secretion.

Molecular weight determination revealed that LG-EPs ranged mainly between 1 × 10⁴ and 1.5 × 10⁴ g/mol, and LC‒MS/MS analysis further revealed the peptide sequences present in LG-EPs, including four peptides with antimicrobial potential and twelve peptides with antioxidant potential. Given that safety is a condition of prior application, LG-EPs were initially tested via the chick embryo allantoic membrane eye irritation assay and erythrocyte hemolysis assay. The findings proved that LG-EPs were highly safe. In vitro functional assessment was performed by investigation of the DPPH radical scavenging, hydroxyl radical scavenging and hyaluronidase inhibition. Taken together, these results reveal that LG-EPs are a good antioxidant and potent hyaluronidase inhibitor and thus could be used in the development of cosmetics or food compounds as antioxidant and anti-inflammatory agents.

Antibacterial assays further support the anti-acne potential of LG-EPs. *C. acnes* is one of the major microorganisms involved in acne pathogenesis, while *S. aureus* is also associated with inflammatory skin disorders and may aggravate cutaneous inflammation. In this study, LG-EPs inhibited both bacteria, with MIC/MBC values of 600 µg/mL against *C. acnes* and MIC/MBC values of 700 µg/mL and 1 mg/mL, respectively, against *S. aureus*. Growth-curve analysis further showed that LG-EPs delayed bacterial proliferation and reduced the final bacterial density. In addition, crystal violet staining demonstrated that LG-EPs significantly inhibited biofilm adhesion in a concentration-dependent manner. Since bacterial proliferation and biofilm formation can promote persistent inflammation and reduce the efficacy of conventional topical treatment, the antibacterial and anti-adhesion effects of LG-EPs provide additional evidence supporting their anti-acne activity. Combined with the peptide-sequence analysis showing antimicrobial potential, these results suggest that LG-EPs may contribute to acne alleviation not only by regulating host inflammatory and sebaceous responses but also by directly suppressing acne-associated bacteria.

LPS is also commonly used to establish models of inflammatory damage. Strong oxidative stresses and powerful inflammatory reactions disrupt barrier functions in the skin and result in the presence of damaged skin that is susceptible to additional inflammation. The PI3K/AKT/mTOR pathway is a major regulator of inflammation and barrier impairment. The results of the ROS scavenging measurements revealed that LG-EPs suppressed oxidative stress conditions by destroying the excess ROS produced during LPS-induced injury. Cellular and molecular assays also revealed that LG-EPs inhibited the activation of the PI3K/AKT/mTOR cascade, hence reducing transcription and the release of proinflammatory cytokines (IL-6, IL-8, and IL-1 in addition to TNF-a) and increasing the expression of barrier-related proteins (AQP3, FLG, and LOR). In vivo experiments were performed on golden hamsters with topical LG-EPs, and no effects on drinking behavior or feeding behavior were detected. Furthermore, the treatment decreased the protrusion of sebaceous glands, and LG-EPs effectively prevented sebum secretion by inhibiting PI3K/AKT/mTOR phosphorylation and reducing the production of lipogenic substrates.

In this paper, the anti-inflammatory and sebum-inhibitory effects of LG-EPs at the cellular, molecular, and animal levels were critically analyzed. The mechanisms of these effects were briefly elucidated, which is helpful in terms of the theoretical and experimental evidence of the use of LG-EPs in anti-acne cosmetic formulations. However, several shortcomings still exist. Research has failed to determine the regulatory effect of LG-EPs on the PI3K/AKT/mTOR signaling pathway at the protein level and its effect on acne-related bacteria. Additionally, the LG-EPs used in this case were not a purified extract; thus, ruling out the potential effects of other components was difficult. Further probing into the structural characteristics of LG-EPs and further clarifying the mechanisms through which it can exert its anti-acne effect are the objectives of future research.

## Conclusion

In this study, the efficacy of LG-EPs was preliminarily examined through biochemical, cellular, molecular, and animal-level experiments, and the results indicated that LG-EPs may exert anti-inflammatory and sebum inhibitory effects by inhibiting the activation of the PI3K/AKT/mTOR signaling pathway and that LG-EPs have the potential to be applied as an anti-acne agent for cosmetic applications.

## Data Availability

The datasets used and/or analysed during the current study are available from the corresponding author on reasonable request.
